# Cell-specific *Eif2b5* mutant mice: novel insights into roles of macroglia in vanishing white matter

**DOI:** 10.1093/brain/awaf171

**Published:** 2025-05-06

**Authors:** Javier Triñanes-Ramos, Marianna Bugiani, Gemma M van Rooijen-van Leeuwen, Juliette A Chevalier, Yuan Jiew Siu, Elise L H van Utenhove, Leoni Hoogterp, Diede Witkamp, Ellen Oudejans, Bastiaan Lodder, Maarten H P Kole, Gesine Saher, Klaus-Armin Nave, Truus E M Abbink, Marjo S van der Knaap

**Affiliations:** Department of Child Neurology, Amsterdam Leukodystrophy Center, Emma Children’s Hospital, Amsterdam University Medical Centers, Amsterdam, 1105 AZ, The Netherlands; Department of Child Neurology, Amsterdam Leukodystrophy Center, Emma Children’s Hospital, Amsterdam University Medical Centers, Amsterdam, 1105 AZ, The Netherlands; Department of Pathology, Amsterdam University Medical Centers, Amsterdam, 1105 AZ, The Netherlands; Department of Child Neurology, Amsterdam Leukodystrophy Center, Emma Children’s Hospital, Amsterdam University Medical Centers, Amsterdam, 1105 AZ, The Netherlands; Department of Child Neurology, Amsterdam Leukodystrophy Center, Emma Children’s Hospital, Amsterdam University Medical Centers, Amsterdam, 1105 AZ, The Netherlands; Department of Child Neurology, Amsterdam Leukodystrophy Center, Emma Children’s Hospital, Amsterdam University Medical Centers, Amsterdam, 1105 AZ, The Netherlands; Department of Child Neurology, Amsterdam Leukodystrophy Center, Emma Children’s Hospital, Amsterdam University Medical Centers, Amsterdam, 1105 AZ, The Netherlands; Department of Child Neurology, Amsterdam Leukodystrophy Center, Emma Children’s Hospital, Amsterdam University Medical Centers, Amsterdam, 1105 AZ, The Netherlands; Department of Child Neurology, Amsterdam Leukodystrophy Center, Emma Children’s Hospital, Amsterdam University Medical Centers, Amsterdam, 1105 AZ, The Netherlands; Department of Child Neurology, Amsterdam Leukodystrophy Center, Emma Children’s Hospital, Amsterdam University Medical Centers, Amsterdam, 1105 AZ, The Netherlands; Axonal Signaling Group, Netherlands Institute for Neurosciences, Royal Netherlands Academy for Arts and Sciences, Amsterdam, 1105 AZ, The Netherlands; Axonal Signaling Group, Netherlands Institute for Neurosciences, Royal Netherlands Academy for Arts and Sciences, Amsterdam, 1105 AZ, The Netherlands; Department of Biology, Faculty of Science, Cell Biology, Neurobiology and Biophysics, Utrecht University, Utrecht, 3584 CH, The Netherlands; Department of Neurogenetics, Max Planck Institute of Experimental Medicine, Göttingen, 37077, Germany; Department of Neurogenetics, Max Planck Institute of Experimental Medicine, Göttingen, 37077, Germany; Department of Child Neurology, Amsterdam Leukodystrophy Center, Emma Children’s Hospital, Amsterdam University Medical Centers, Amsterdam, 1105 AZ, The Netherlands; Department of Child Neurology, Amsterdam Leukodystrophy Center, Emma Children’s Hospital, Amsterdam University Medical Centers, Amsterdam, 1105 AZ, The Netherlands

**Keywords:** vanishing white matter, glia, conditional mice

## Abstract

Vanishing white matter (VWM) is a leukodystrophy caused by mutations in any of the genes encoding the subunits of the eukaryotic translation initiation factor 2B (eIF2B), a central factor in mRNA translation initiation and regulator of the translation rate during the integrated stress response. Clinically, VWM is characterized by chronic motor and cognitive decline and premature death. Neuropathology shows selective white matter involvement with dysmorphic, immature astrocytes and defective reactive astrogliosis, while oligodendrocytes show increased expression of immaturity and proliferation markers and neurons look normal. Expression of the ATF4 transcriptome is increased in the white matter. These characteristics have been replicated successfully in eIF2B mutant mouse models. Until now, the relative contribution of each cell type to the development of VWM has remained unclear. Understanding the vulnerability of specific cell types for VWM is crucial for understanding disease mechanisms and developing effective therapies.

We generated astrocyte-, oligodendrocyte- and neuron-specific *Eif2b5* conditional mouse lines to determine the role of each mutant cell type in the onset and development of the disease. We evaluated motor performance, white matter pathology and the expression of myelin-related proteins. We analysed astrocyte and oligodendrocyte density, maturity, morphology, proliferation and apoptosis. We investigated the expression of the integrated stress response-/ATF4-related genes and proteins, in addition to their localization within the glial cells.

At age 9 months, we found that astrocyte-specific *Eif2b5* conditional mice showed very mild ataxia, extensive intramyelinic vacuolization, normal density and maturity of oligodendrocytes, and high expression of ATF4-related genes. Oligodendrocyte-specific *Eif2b5* conditional mice developed gait ataxia that matched the phenotype of the whole-body *Eif2b5* mutant line. Myelin looked normal, but numerous axons were unmyelinated; astrocytes were reactive, and oligodendrocytes were immature and in cell cycle. The enhanced ATF4 transcriptome was minor compared with the astrocyte-specific lines. The neuron-specific *Eif2b5* conditional line exhibited a very mild phenotype and none of the major characteristics of VWM.

Our findings highlight a complex effect of *Eif2b5* mutations in different brain cell types leading to the clinical and neuropathological characteristics of VWM. Oligodendrocytes are the major contributors to the development of ataxia, but astrocyte-specific *Eif2b5* conditional mice display several histological and molecular key features of VWM. In conclusion, the view of a single cell population being responsible for the onset and development of VWM needs to be replaced by the concept of VWM as a disease involving diverse cell types.

## Teaser

Vanishing white matter (VWM) is a leukodystrophy caused by a genetic defect in translation initiation, with astrocytes thought to play a key role. Triñanes-Ramos *et al.* use conditional *Eif2b5* mouse models to assess the individual contributions of oligodendrocytes, astrocytes and neurons, and reveal complex interactions among these cell types in VWM.

## Introduction

Vanishing white matter (VWM; OMIM 306896) is a leukodystrophy clinically characterized by chronic motor and cognitive decline. In addition, episodes of major deterioration occur, triggered by stressors such as febrile infections or head trauma.^1,2^ The disease is caused by pathogenic variants in any of the genes *EIF2B1*–*EIF2B5*, encoding the α- to ε-subunits of eukaryotic initiation factor 2B (eIF2B).^3,4^ eIF2B is a guanine nucleotide exchange factor essential for initiation of mRNA translation. It catalyses the conversion of inactive eIF2•GDP to active eIF2•GTP, allowing the formation of the ternary complex eIF2•GTP-methionyl-tRNA, which brings the first amino to the ribosome.^5^ eIF2B is central in tuning the levels of protein synthesis in stress conditions and plays a pivotal role in the integrated stress response (ISR). The ISR is an adaptive pathway intended to reduce global mRNA translation while activating the translation of specific mRNAs, such as transcription factor ATF4.^6^ During the ISR, eIF2B is inhibited, attenuating global protein synthesis and activation of the ATF4 transcriptome.^6^ Mutant eIF2B has decreased activity.^7^ ISR dysregulation with constitutively increased levels of ATF4 and ATF4-driven factors is characteristic of VWM patient brains.^8,9^

In VWM patients, histopathology shows white matter rarefaction and cystic degeneration.^2,10^ There is a lack of myelin, and the myelin present is often vacuolated. Reactive astrogliosis is meagre, and astrocytes show dysmorphic and immature characteristics, with blunt processes instead of their typical fine branching structure.^10-12^ Oligodendrocytes have an increased density and show an increased expression of immaturity and proliferation markers.^11,13^ In contrast to the prominent glial pathology, neurons are a much less affected cell type in VWM, although not completely normal.^14,15^

Previously described mouse models for VWM replicate key characteristics of the human disease, including ataxia, major astrocytic and oligodendrocyte abnormalities^16^ and minor neuronal abnormalities.^15^ These models have been advantageous in exploring the glial pathology^16^ and the dysregulated ISR in VWM.^8^

It is remarkable that eIF2B is a ubiquitous housekeeping factor, whereas VWM manifests predominantly in the white matter of the brain, mainly affecting astrocytes and oligodendrocytes.^1,2,10,11^ This remarkable selective vulnerability has been the focus of research.^11,16,17^ Previous findings have suggested that astrocyte pathology is primary and that the pathology of oligodendrocytes^16^ and neurons^15^ is secondary. Our findings also suggested that ISR dysregulation involves astrocytes selectively.^8,18^

In the present study, we used the previously described *Eif2b5* mouse model (c.572G>A; p.Arg191His)^16^ as a premise to generate astrocyte-, oligodendrocyte- and neuron-specific *Eif2b5* mouse mutant lines in an endeavour to dissect cellular involvement in VWM pathology.

## Materials and methods

### Conditional VWM mutant mice

**Figure 1:**
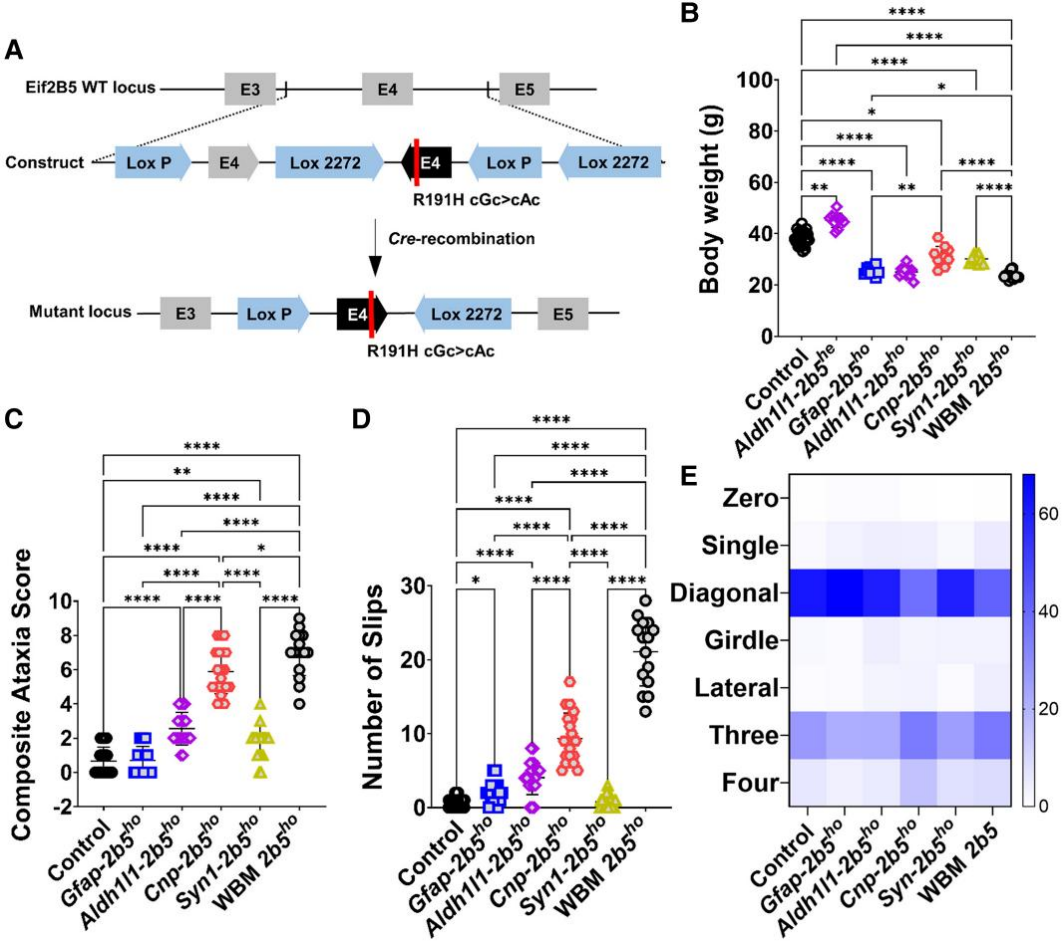
**Generation and motor evaluation of the mouse models.** (**A**) *Eifb5* conditional mice were generated by providing the exon 4 with a Flex Cre-switch that incorporates a conditional point mutation (R191H). Those cells expressing Cre (astrocytes, oligodendrocytes or neurons) will have recombination across the loxP sites: the wild-type exon 4 is spliced out, and the exon 4 with the desired point mutation (R191H) is inserted, resulting in expression of the mutant *Eif2b5*. E3: exon 3, E4: exon 4, E5: exon 5. (**B**) Body weight of male mice at the end of the experiment, at 9 months of age. Control group *n* = 40 [8 wild-type (WT), 11 *Gfap-2b5^he^*, 10 *Cnp-2b5^he^* and 11 *Syn-2b5^he^*], each conditional *2b5* group *n* = 10, and WBM-*2b5^ho^ n* = 16. (**C**) Composite ataxia score of all mice (males and females) at 9 months of age. Control group *n* = 80 (8 WT, 18 *Gfap-2b5^he^*, 18 *Aldh1l1-2b5^he^*, 17 *Cnp-2b5^he^* and 19 *Syn-2b5^he^*), each conditional *2b5^ho^* group *n* = 20, and WBM-*2b5^ho^ n* = 16. (**D**) At 9 months of age, all mice traversed a balance beam three times. The number of slips recorded during the last run is shown in the graph. Control group *n* = 80 (8 WT, 18 *Gfap-2b5^he^*, 18 *Aldh1l1-2b5^he^*, 17 *Cnp-2b5^he^* and 19 *Syn-2b5^he^*), for each conditional *2b5^ho^* group *n* = 20, and WBM-*2b5^ho^ n* = 16. (**E**) Heat map indicating the percentage of the time that animals used the different types of supports during the CatWalk XT test. Control group *n* = 80 (8 WT, 18 *Gfap-2b5^he^*, 18 *Aldh1l1-2b5^he^*, 17 *Cnp-2b5^he^* and 19 *Syn-2b5^he^*), for each conditional *2b5^ho^* group *n* = 20, and WBM-*2b5^ho^ n* = 16. *P < 0.05, **P < 0.01, ****P < 0.0001.

We previously developed *Eif2b5* mutant mice (c.572G>A; p.Arg191His) that in the homozygous state (*2b5^ho^* mice) replicate key characteristics of human VWM.^16^ Heterozygous *Eif2b5* mutant mice (*2b5^he^* mice) have no phenotype, as expected for a recessive disease.^16^ The *Eif2b5* mutant mouse served as the basis for generating new conditional mutant mice (*Eif2b5^flox^*). In the *Eif2b5^flox^* mice, wild-type exon 4 is annexed to mutant exon 4 in the antisense direction (Fig. 1A). Crossing the *Eif2b5^flox^* mouse with a Cre line results in the recombination across the loxP sites, leading to deletion of the wild-type exon 4 and inversion of the mutant exon 4 with the desired mutation (Arg191His) (Fig. 1A and Supplementary Fig. 1A–I). Conditional *Eif2b5* mutant mice were created by crossing the *Eif2b5^flox^* mice with *Cnp-*^19^ (*Cnp-2b5*), *Gfap-*^20^ (*Gfap-2b5*) and *Syn1-*^21^ (*Syn1-2b5*) driven Cre recombinases. Additionally, we used a previously developed *Aldh1l1-*driven CreERT2 recombinase^22^ (*Aldh1l1-2b5*) to confirm the results obtained in the *Gfap-2b5* line. In the *Aldh1l1-2b5* line, the activation of recombination was done by five consecutive daily tamoxifen injections on postnatal days 21–25. The cell specificity of Cre expression was evaluated at the end of the experiment by immunostaining (Supplementary Fig. 2).

To reduce the number of experimental animals, we compared the locomotor parameters of the *Eif2b5* conditional mice with the same parameters obtained in wild-type (WT) and whole-body mutant (WBM) *2b5^ho^* mice bred for other contemporaneous studies,^18^ and locomotor parameters were measured in the same conditions and laboratories and with the same equipment. Tissue from WT and WBM *2b5^ho^* mice from our biobank was included for the histological and molecular comparisons. Conditional heterozygous mice were included in the control group, if statistical tests showed no differences from WT mice. In all conditional lines, we did not find sex differences for motor deterioration and histopathology and therefore included the same number of males and females for each conditional line. WT and WBM-*2b5^ho^* groups contained only male animals. Body weight comparison, western blot and gene-expression analyses were therefore performed only on male mice for all groups. All genotypes were determined by PCR; germline recombination was not detected in any of the experimental animals (Supplementary Fig. 1A–C).

All animals were weaned around postnatal day 21, group housed, and maintained in a 12 h–12 h light–dark cycle with *ad libitum* access to water and food. All mice were sacrificed when they reached 9 months of age, immediately after all motor evaluations. Animal experiments were performed in compliance with the Dutch and European law and with approval of the local animal care and use committee of the Vrije Universiteit and Amsterdam Medical Center [licenses FGA13-02, CCD AVD1120020172804 and AVD11800202317078; work protocols 2804-NEU15, NEUV17-2804-1-01 and NEUV23-17078-2-05].

### Motor evaluation

Between 4 and 9 months of age, all mice were evaluated weekly. Ledge test, hindlimb clasping, gait, and pelvic tilt were scored from zero (not affected) to three (severely affected), then added up to produce the composite ataxia score (CAS).^23,24^ Kyphosis was scored separately from zero to three.^24^ At the end of the evaluation period, motor coordination was assessed on a balance beam, and a complete gait analysis was performed on a CatWalk XT.^18^ The image and data analysis was performed by researchers blinded to the genotype of the animals. CatWalk variables were categorized as previously described.^25^ To determine which specific variables were changed in VWM, 60 different variables were compared between male WBM-*2b5* mice and heterozygous control males. Spatial variables^25^ were excluded from the analysis for being highly dependent on body weight. Thirty-two variables exhibited statistically significant differences between control and WBM-*2b5^ho^* mice and were defined as VWM parameters (Supplementary Tables 1 and 2). These VWM parameters showed no difference between males and females within the same line, and therefore both sexes were included in the study.

### Gene expression analysis by quantitative PCR

Cerebella from 9-month-old conditional mice and 6-month-old WT and WBM-*2b5^ho^* mice were processed as previously described.^26^ Tissues from 6-month-old WT and WBM-*2b5^ho^* were considered adequate as controls based on previous research showing that from the age of 4 months until the humane end point, WBM-*2b5^ho^* mice have increased numbers of unmyelinated axons^15,16^ and sustained upregulation of the ATF4 transcriptome.^8^ Six-month-old WT and 9-month-old conditional heterozygous mice were grouped as controls because no differences were found between them.

Cerebella were divided into two fractions for RNA and protein extraction. Total RNA was extracted from one of these fractions with Trizol™ reagent (Invitrogen). Reverse transcription and quantitative PCR (qPCR) were performed as described previously.^8^ Primers were designed using PrimerBLAST. *Hprt* mRNA was used as reference. All primers used are listed in Supplementary Table 3.

### Western blotting

Total protein obtained from cerebella was determined by the Bradford method. Fifty micrograms of total protein was loaded into SDS-PAGE and transferred onto polyvinylidene difluoride membranes. Protein loading was checked with 2,2,2-trichloroethanol. Membranes were blocked with 5% non-fat dry milk and incubated overnight at 4°C with primary antibodies against PLP/DM20, MBP and 4E-BP1 (Supplementary Table 4). Horseradish peroxidase-linked secondary antibodies were used to develop the immunoblots, and the signal was detected with enhanced chemiluminescent substrate (SuperSignal West Femto Substrate, Fisher Scientific). Images were taken with the Bio-Rad ChemiDoc imaging system and analysed with ImageJ. Potential technical variation among immunoblots was resolved using factor correction.^27^

### Histology, immunofluorescence, immunohistochemistry and electron microscopy

For this purpose, all (the WT, WBM and conditional) mice were terminated at 9 months of age. They were anaesthetized with tribromoethanol (Avertin) or pentobarbital and perfused with 4% paraformaldehyde. Brains were maintained in paraformaldehyde for 24–48 h and conserved in 70% ethanol until paraffin embedding. Sagittally cut brain sections (5–6 μm thick) were deparaffinized and rehydrated. For immunofluorescence, samples were incubated in 0.1 m glycine to quench autofluorescence attributable to aldehyde groups, and heat-induced antigen retrieval was performed. The tissue was then permeabilized in 0.1% Triton X-100 and blocked with 3% bovine serum albumin and mouse-on-mouse blocking buffer when primary mouse antibodies were used. Primary antibodies (Supplementary Table 4) were incubated overnight at room temperature. Secondary antibodies (Alexa Fluor) were incubated 2 h at room temperature. Sudan Black (0.1%, in 70% ethanol) was used to quench autofluorescence, and 4′,6-diamidino-2-phenylindol (DAPI) was used for nuclear counterstaining. For immunohistochemistry with diaminobenzidine, an extra step of endogenous peroxidase inhibition was performed after rehydration by incubating the sections in PBS containing 3% H_2_O_2_. Horseradish peroxidase-linked secondary antibodies were incubated for 2 h at room temperature with diaminobenzidine as substrate. Haematoxylin was used as counterstain. All sections were examined using a Leica DM500; nuclear localization of Ki67 was studied using a Leica SP8 confocal microscope. PLP/DM20 staining was used to determine the intramyelinic vacuoles. GFAP^+^ cells, GFAP^+^SOX9^+^ cells, S100β^+^ cells and SOX10^+^ nuclei were expressed as a percentage of positive nuclei or cells against the total number of DAPI^+^ nuclei. Cycling cells were detected by staining against Ki67 and quantified as the percentage of cycling oligodendrocytes (Ki67^+^SOX10^+^/SOX10^+^ × 100) and the percentage of cycling astrocytes (Ki67^+^GFAP^+^/GFAP^+^ × 100). Cell death was assessed by both caspase-3 staining and TUNEL assay (4812-30-K, R&D systems). Immature astrocytes were identified and quantified by nestin^+^GFAP^+^ staining and expressed as a percentage of total GFAP^+^ cells (nestin^+^GFAP^+^/GFAP^+^ × 100). Bergmann glia translocation was evaluated by staining against S100β, as described.^16,17^ ISR dysregulation was determined by expression of ATF4-regulated eukaryotic translation initiation factor 4E binding protein-1 (4E-BP1). 4E-BP1-positive astrocytes were identified by co-staining with GFAP, and 4E-BP1-positive oligodendrocytes by co-staining with OLIG2.

For electron microscopy, samples were OCT embedded and processed as previously described.^28^ Pictures were taken with a Talos L120C at 120 kV with a Ceta 16M camera.

### Sholl analysis in astrocytes

Astrocyte morphology was assessed on sections stained for GFAP. Five to seven GFAP-stained astrocytes in the corpus callosum per condition and mouse were drawn using the paintbrush tool in ImageJ. Sholl analysis was performed with a starting radius of 10 μm and radius steps of 5 μm. For each radius step, the number of astrocyte processes crossing a circle was recorded as the number of intersections. For each animal, the total number of intersections per astrocyte were averaged.

### Statistical analysis

Statistical analyses were performed in GraphPad Prism 9 for CAS, balance beam, histological and molecular data and R for CatWalk XT data.^18^ Differences were considered statistically significant when *P* < 0.05. Normality of the data sets was tested by the Shapiro–Wilk test. If the variable passed the normality test, the differences among all groups were assessed with one-way ANOVA and multiple comparisons were performed by Tukey's *post hoc* test. If the distribution of the variable was not normal, differences were assessed by Kruskal–Wallis test with Dunn's multiple comparisons. Two-way ANOVA followed by Tukey's *post hoc* test was used for the Sholl analysis (genotype and radius) and when analysing different isoforms of a protein in a western blot (genotype and isoform). Details about numbers and sex of the animals in each test are provided in the figure legends.

## Results

### Comparison of control mice

WT and all conditional *2b5^he^* animals did not display differences in motor tests, neuropathology or molecular analysis. Therefore, they were grouped together. The only exception was the body weight of *Aldh1l1-2b5^he^* mice, which was higher than in all other control groups (45.25 ± 0.14 versus 38.52 ± 2.68 g, *P* < 0.0001; Fig. 1B and Supplementary Fig. 3A and B) and therefore considered separately.

### Growth, coordination and gait

Astrocyte-specific *Gfap-2b5^ho^* mice had a body weight lower than control animals (25.54 ± 1.56 versus 38.44 ± 2.62 g, *P* < 0.0001) and comparable to WBM-*2b5^ho^* mice (23.65 ± 1.34 g) (Fig. 1B and Supplementary Fig. 3A and B). *Gfap-2b5^ho^* mice did not develop gait ataxia, with only hindlimb clasping and the ledge test contributing to their CAS at later disease stage (Fig. 1C and Supplementary Fig. 3C–G). *Gfap-2b5^ho^* mice developed kyphosis, which was not observed in control mice, including *Gfap-2b5^he^* animals (Supplementary Fig. 3H), and was not reported in previous VWM mouse models.^8,18,24,29^ When analysing motor coordination on the balance beam, *Gfap-2b5^ho^* mice had slightly more slips than control mice (2.1 ± 0.33 versus 0.56 ± 0.76 slips, *P* < 0.05; Fig. 1D). A complete gait analysis with CatWalk XT determined that *Gfap-2b5^ho^* animals differed from controls in 2 of the 32 variables defined as VWM parameters (Supplementary Table 1), while being different for all parameters compared with WBM-*2b5^ho^* mice (Supplementary Table 2). Regarding paw combinations used for walking support, most of the time the *Gfap-2b5^ho^* animals walked with diagonal paw pairs contacting the plate (diagonal support) (68.16% ± 10.38%), similar to controls (62.22% ± 13.35%) and more than WBM-*2b5^ho^* mice (41.95% ± 9.33%; *P* < 0.001) (Fig. 1E and Supplementary Tables 1 and 2).

*Aldh1l1-2b5^ho^* mice showed a body weight similar to *Gfap-2b5^ho^* and WBM-*2b5^ho^* mice, different from controls (25.01 ± 2.25 versus 38.44 ± 2.62 g, *P* < 0.0001; Fig. 1B and Supplementary Fig. 3A). The CAS for *Aldh1l1-2b5^ho^* animals at week 36 (2.55 ± 0.94) was higher than for controls (0.66 ± 0.81, *P* < 0.0001) and *Gfap-2b5^ho^* mice (0.7 ± 0.8, *P* < 0.0001) and significantly lower than for WBM-*2b5^ho^* mice (6.97 ± 1.31, *P* < 0.0001) (Fig. 1C). *Aldh1l1-2b5^ho^* mice did not show gait ataxia: their CAS was increased owing to a worse hindlimb clasping and poor performance in the ledge test (Supplementary Fig. 3C–G). *Aldh1l1-2b5^ho^* animals developed a higher degree of kyphosis than *Gfap-2b5^ho^* animals (Supplementary Fig. 3H), a feature seen only in the astrocyte-specific mutant lines. *Aldh1l1-2b5^ho^* mice showed higher number of slips on the balance beam than control animals (4.05 ± 2.28 versus 0.56 ± 0.76 slips, *P* < 0.0001), similar to *Gfap-2b5^ho^* and lower than WBM-*2b5^ho^* mice (21.07 ± 4.60, *P* < 0.0001) (Fig. 1D). The CatWalk XT analysis showed that *Aldh1l1-2b5^ho^* mice are somewhat more affected than *Gfap-2b5^ho^* animals, with 8 of 32 VWM parameters different from those in control mice (Supplementary Table 1) and with 25 VWM parameters different from those in WBM-*2b5^ho^* mice (Supplementary Table 2). *Aldh1l1-2b5^ho^* mice used a similar diagonal support to controls (61.54% ± 14.22% versus 62.22% ± 13.35%), more than WBM-*2b5^ho^* mice (41.95% ± 9.33%; *P* < 0.001) (Fig. 1E and Supplementary Tables 1 and 2).

Oligodendrocyte-specific *Cnp-2b5^ho^* mice had lower body weight than controls (32.12 ± 3.87 versus 38.44 ± 2.62 g, *P* < 0.05), but higher than *Gfap-2b5^ho^* (25.54 ± 1.56 g, *P* < 0.01) and WBM-*2b5^ho^* mice (23.65 ± 1.56 g, *P* < 0.0001) (Fig. 1B and Supplementary Fig. 3A and 3B). *Cnp-2b5^ho^* mice had a higher CAS at week 36 than any other homozygous conditional mouse line, but lower than WBM-*2b5^ho^* mice (5.90 ± 1.30 versus 6.97 ± 1.31, *P* < 0.05; Fig. 1C). The development of ataxia started earlier in the *Cnp-2b5^ho^* line, but the decline was slower than in WBM-*2b5^ho^* mice (Supplementary Fig. 3C–G). On the balance beam, *Cnp-2b5^ho^* mice had more slips than any other conditional mutant line but fewer than WBM-*2b5^ho^* mice (9.35 ± 3.42 versus 21.07 ± 4.60, *P* < 0.0001; Fig. 1D). A complete gait analysis revealed that *Cnp-2b5^ho^* animals had the same phenotype as WBM-*2b5^ho^* mice, with all VWM parameters different from those in control mice (Supplementary Fig. 3I and Supplementary Table 1). In comparison to WBM-*2b5^ho^* mice, *Cnp-2b5^ho^* animals showed similar values in 27 VWM parameters and worse values in the other 5 (Supplementary Table 2). *Cnp-2b5^ho^* mice showed a reduction in diagonal support compared with controls (38.13% ± 13.40% versus 62.22% ± 13.35%; *P* < 0.001), similar to WBM-*2b5^ho^* animals (41.95% ± 9.33%). The reduction in diagonal support was compensated for by an increase in all other types of support (Fig. 1E and Supplementary Tables 1 and 2).

The *Syn1-2b5^ho^* mice had a lower body weight than controls (30.19 ± 1.68 versus 38.44 ± 2.62 g, *P* < 0.0001), but higher than both WBM-*2b5^ho^* (23.65 ± 1.34 g, *P* < 0.0001) and astrocyte-specific conditional lines and similar to *Cnp-2b5^ho^* mice (Fig. 1B and Supplementary Fig. 3A and B). At week 36, the CAS of *Syn1-2b5^ho^* animals was higher than that of controls (1.72 ± 0.96 versus 0.66 ± 0.81, *P* < 0.0001), but lower than that of WBM-*2b5^ho^* mice (6.98 ± 1.31, *P* < 0.0001). The CAS of the *Syn1-2b5^ho^* animals in the last week was similar to that of *Aldh1l1-2b5^ho^* mice, but higher than that of *Gfap-2b5^ho^* mice (1.72 ± 0.96 versus 0.70 ± 0.80, *P* < 0.05) and lower than that of *Cnp-2b5^ho^* mice (5.90 ± 1.30, *P* < 0.0001) (Fig. 1C). Regarding CAS parameters, the *Syn1-2b5^ho^* line displayed moderate pelvic tilt and some problems with the ledge test (Supplementary Fig. 3C–G). On the balance beam, the performance of the *Syn1-2b5^ho^* animals was similar to that of controls. The CatWalk XT analysis showed that *Syn1-2b5^ho^* animals were least affected, with only 3 VWM parameters different from those in controls (Supplementary Table 1) and the other 29 parameters different from those in WBM-*2b5^ho^* animals (Supplementary Table 2). As in control mice, diagonal support was used most of the time by *Syn1-2b5^ho^* mice (59.68% ± 13.26%; Fig. 1E and Supplementary Tables 1 and 2).

### White matter

**Figure 2:**
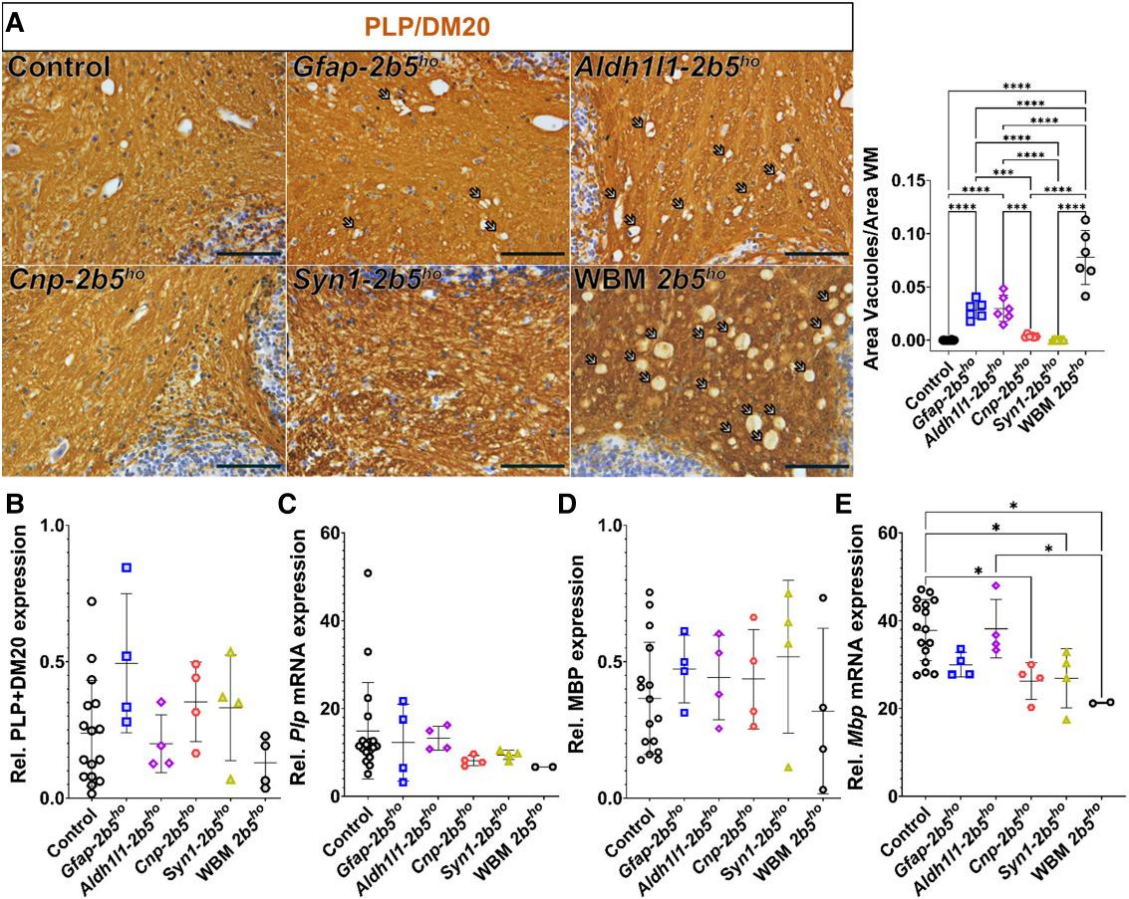
**Evaluation of myelin.** (**A**) All mice were 9 months old for this study. Sagittally cut brain sections from 24 conditional heterozygous mice (six per conditional line used as controls), six conditional homozygous mice per mutant line and six WBM-*2b5^ho^* were stained against PLP/DM20. The image shows representative sections of the cerebellar white matter for each condition. Arrows indicate the presence of intramyelinic vacuoles. All groups contain 50% of each sex. The *right panel* shows quantification of the proportion of vacuolated area per total area of the white matter (WM) in the cerebellum. (**B**–**E**) For this study, heterozygous and homozygous conditional mice were 9 months old, whereas wild-type and WBM-*2b5^ho^* mice were 6 months old. (**B**) Cerebella from 16 heterozygous control mice, four homozygous conditional mice per line and four WBM-*2b5^ho^* mice were used to determine levels of PLP/DM20 expression by western blot. All mice used for this purpose were males. (**C**) Cerebella from 16 heterozygous control mice, four homozygous conditional mice per line and two WBM-*2b5^ho^* mice were used for quantification of *Plp* mRNA in the cerebellum. All males. (**D**) Quantification of MBP expression in the cerebellum by western blot. Same animals as in **B**. (**E**) Quantification of *Mbp* mRNA in the cerebellum. Same animals as in **C**. Sample size for western blot and quantitative PCR analysis is consistent with prior studies.^8,18^ Scale bar: 100 μm. *P < 0.05, ***P < 0.001, ****P < 0.0001.

*Gfap-2b5^ho^* animals showed positive PLP immunohistochemistry in all white matter structures (Fig. 2 and Supplementary Fig. 4A). PLP staining showed intramyelinic vacuoles in all *Gfap-2b5^ho^* animals, although much less than in WBM-*2b5^ho^* mice (Fig. 2A). Intramyelinic vacuoles were not observed in control mice (Fig. 2A). Western blot analyses of cerebellar PLP/DM20 and MBP showed a large variability among individuals (Supplementary Fig. 4B), with no differences in the expression levels of these myelin-related proteins (Fig. 2B and D). qPCR to quantify *Plp* mRNA showed that the expression in *Gfap-2b5^ho^* mice was not significantly different from that in any of the other mice (Fig. 2C). qPCR to quantify all mRNA variants of the *Mbp* gene showed that the expression in *Gfap-2b5^ho^* mice (29.98 ± 2.79) was in between that of controls (37.84 ± 6.94) and WBM-*2b5^ho^* (21.31 ± 0.19) animals, but not significantly different from either of them (Fig. 2E).

**Figure 3:**
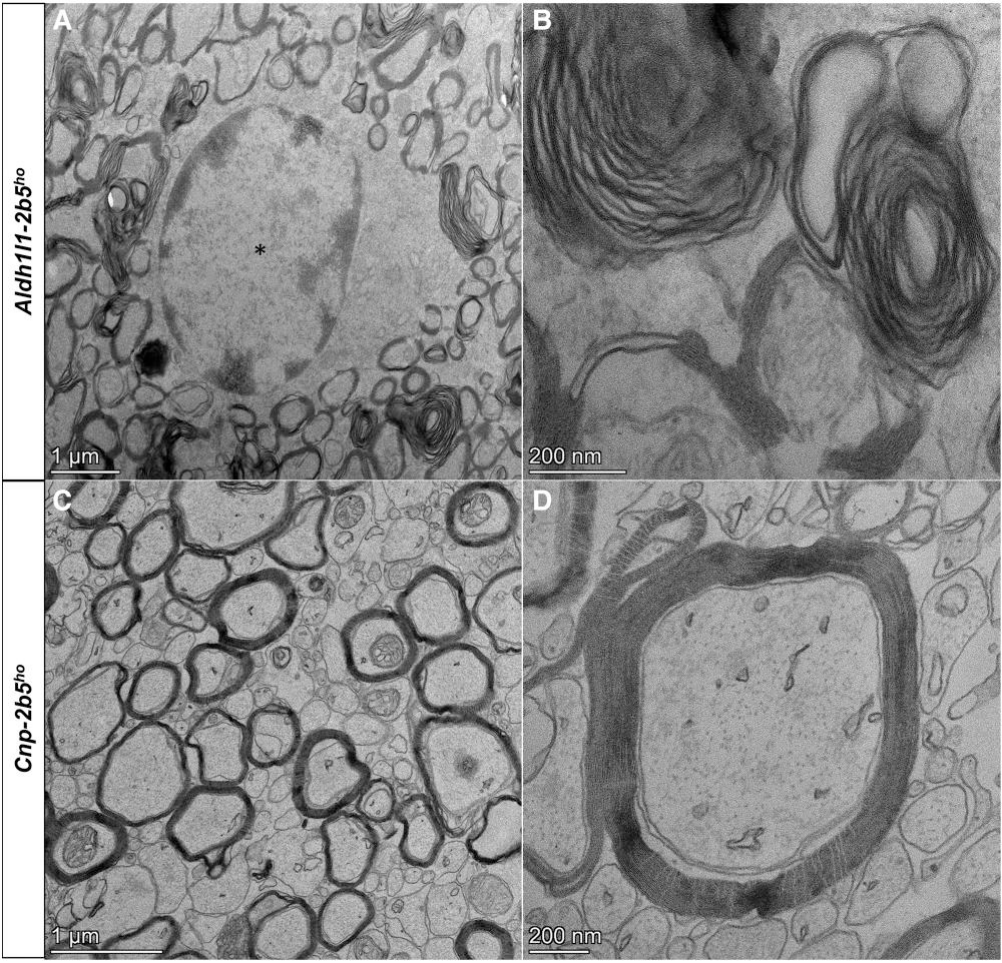
**Myelin ultrastructure in *Aldh1l1*- and *Cnp*-*2b5^ho^* mice.** Ultrastructural examination of sagittal sections of the splenium of the corpus callosum of a conditional *Aldh1l1-2b5^ho^* and a conditional *Cnp-2b5^ho^* mouse, both 9 months old. (**A**) Image of the *Adh1l1-2b5^ho^* mouse, showing a central astrocyte (asterisk) surrounded by several axons with intramyelinic vacuoles. (**B**) At higher magnification, the myelin sheaths appear uncompact, with fluid between the lamellae. (**C**) Image of the *Cnp-2b5^ho^* mouse, showing the presence of numerous unmyelinated axons. (**D**) At higher magnification, myelinated axons show a normal myelin structure and compaction.

In *Aldh1l1-2b5^ho^* mice, PLP and MBP staining showed the presence of mature myelin in all white matter structures (Supplementary Fig. 4A), but with a higher degree of intramyelinic vacuolization than *Gfap-2b5^ho^* animals, although still lower than WBM-*2b5^ho^* (Fig. 2A). Western blot analyses of the cerebella of *Aldh1l1-2b5^ho^* mice showed also a high inter-individual variability of PLP/DM20 and MBP levels (Supplementary Fig. 4B) and no differences from any other group (Fig. 2B and D). *Aldh1l1-2b5^ho^* mice had normal *Plp* and *Mbp* mRNA levels in the cerebellum (Fig. 2C and E). Electron microscopy of an *Aldh1l1-2b5^ho^* mouse confirmed extensive intramyelinic vacuolization in the corpus callosum (Fig. 3).

*Cnp-2b5^ho^* mice showed PLP immunostaining similar to control mice, with absence of intramyelinic vacuoles (Fig. 2A and Supplementary Fig. 4A). *Cnp-2b5^ho^* mice also showed high inter-individual variability in the western blot levels of PLP/DM20 and MBP (Supplementary Fig. 4B), with no differences from other animals (Fig. 2B and D). *Plp* mRNA levels in *Cnp-2b5^ho^* mice were normal (Fig. 2C), whereas *Mbp* mRNA levels were lower than those in control mice (26.25 ± 4.19 versus 37.84 ± 6.94; *P* < 0.05; Fig. 2E). Electron microscopy of a *Cnp-2b5^ho^* mouse revealed increased numbers of unmyelinated axons in the corpus callosum, but otherwise myelin sheaths looked normal (Fig. 3).

In *Syn1-2b5^ho^* mice, immunostaining against PLP was similar to control mice, with absence of intramyelinic vacuoles (Fig. 2A and Supplementary Fig. 4A). Analysis of PLP and MBP by western blot in the cerebellum of *Syn1-2b5^ho^* mice showed the same inter-individual variability (Fig. 2B and D and Supplementary Fig. 4B). *Syn1-2b5^ho^* mice had normal *Plp* (Fig. 2C) and lower *Mbp* mRNA levels in the cerebellum than control mice (26.86 ± 6.74 versus 37.84 ± 6.94; *P* < 0.05; Fig. 2E).

### Oligodendrocytes

**Figure 4:**
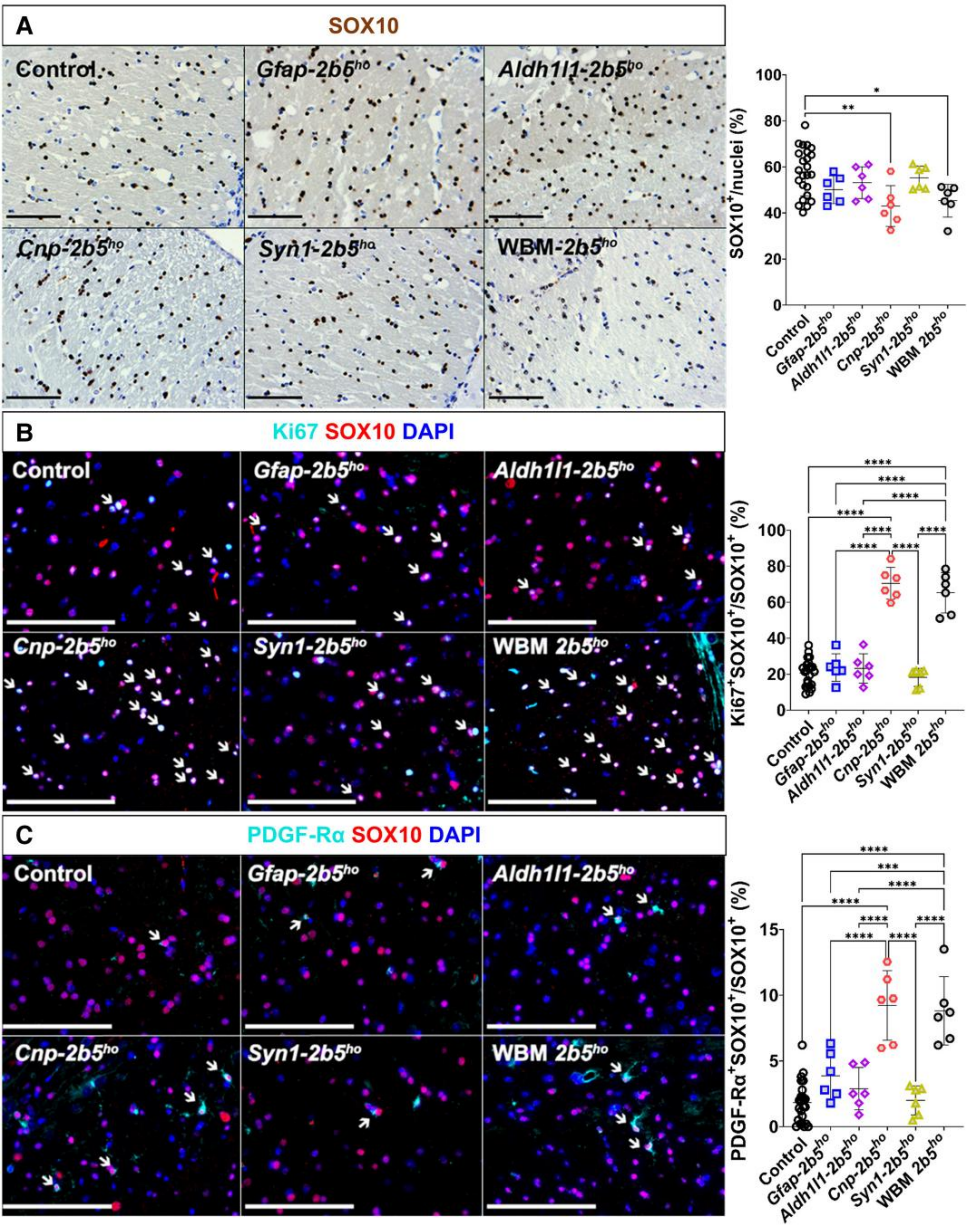
**Density and maturation of oligodendrocytes.** All mice were 9 months old for this study. Sagittally cut brain sections through the corpus callosum from 24 conditional heterozygous mice (six per conditional line, used as controls), six conditional homozygous mice per mutant line and six WBM-*2b5^ho^* mice were stained against: (**A**) SOX10 (diaminobenzidine); (**B**) Ki67 (cyan) and SOX10 (red) counterstained with DAPI (arrows indicate those cells in which both factors are co-expressed); and (**C**) PDGF-Rα (cyan) and SOX10 (red) counterstained with DAPI (arrows indicate those cells in which both factors are co-expressed). The corresponding quantification is shown on the *right* side of each panel. *P < 0.05, **P < 0.01, ***P < 0.001, ****P < 0.0001.

*Gfap-2b5^ho^* mice showed a percentage of SOX10^+^ cells in the corpus callosum (50.17% ± 6.01%) in the range of controls (57.88% ± 10.35%) and WBM-*2b5^ho^* mice (45.32% ± 7.12%) (Fig. 4A)*. Gfap-2b5^ho^* mice showed a similar percentage of Ki67^+^SOX10^+^ oligodendrocytes to controls (23.72% ± 7.61% versus 20.71% ± 7.34%), lower than WBM-*2b5^ho^* mice (65.27% ± 11.23%, *P* < 0.0001) (Fig. 4B). Cell death was not detected in *Gfap-2b5^ho^* mice (Supplementary Fig. 5A–F). The percentage of PDGF-Rα^+^ oligodendrocytes in the corpus callosum of *Gfap-2b5^ho^* mice was similar to that in controls (3.86% ± 1.80% versus 1.84% ± 1.57%) and lower than in VWM *2b5^ho^* mice (8.80% ± 2.60%, *P* < 0.0001) (Fig. 4C).

Oligodendrocytes in the *Aldh1l1-2b5^ho^* mice had similar characteristics to the *Gfap-2b5^ho^* animals. The percentage of SOX10^+^ cells (53.17% ± 6.91%) was not significantly different from control (57.88% ± 10.35%) and WBM-*2b5^ho^* (45.32% ± 7.12%) mice, although closer to the latter (Fig. 4A), and the percentage of Ki67^+^ oligodendrocytes (23.22% ± 8.11%) (Fig. 4B) and PDGF-Rα^+^ oligodendrocytes (2.90% ± 1.61%) (Fig. 4C) in the corpus callosum was similar to controls and significantly reduced in comparison to WBM-*2b5^ho^* mice (8.80% ± 2.60%, *P* < 0.0001). Cell death was not detected (Supplementary Fig. 5A–F).

Oligodendrocytes from the *Cnp-2b5^ho^* mice were most similar to those of the WBM-*2b5^ho^* line. *Cnp-2b5^ho^* animals had lower density of SOX10^+^ cells in the corpus callosum than controls (42.98% ± 8.88% versus 57.88% ± 10.35%; *P* < 0.0001) and similar to WBM-*2b5^ho^* mice (45.32% ± 7.12%) (Fig. 4B). *Cnp-2b5^ho^* mice had a higher percentage of Ki67^+^ oligodendrocytes than controls (70.49% ± 8.82% versus 20.71% ± 0.34%; *P* < 0.0001) or any other conditional line, and similar to the WBM-*2b5^ho^* animals (65.27% ± 11.23%) (Fig. 4B). In both mouse lines, SOX10^+^ cells showed a homogeneous Ki67 nuclear staining, whereas in SOX10^−^ cells, Ki67, when present, was localized in the heterochromatin (Supplementary Fig. 5G). The homogeneous Ki67 pattern together with the low density of CyclinD1^+^ cells in the corpus callosum of *Cnp-2b5^ho^* mice (Supplementary Fig. 5H), similar to WBM-*2b5^ho^* mice (Supplementary Fig. 5I), indicated that most of those SOX10^+^Ki67^+^ cells could be in the S phase of the cell cycle. Cell death was not detected in any *Cnp-2b5^ho^* animal (Supplementary Fig. 5A–F). The percentage of PDGF-Rα^+^ oligodendrocytes was also higher in *Cnp-2b5^ho^* animals than in controls (9.22% ± 2.64% versus 1.83% ± 1.57%; *P* < 0.0001) or any other conditional mutant line, and similar to the percentage seen in the WBM-*2b5^ho^* mice (8.80% ± 2.60%) (Fig. 4C).

Oligodendrocytes of the *Syn1-2b5^ho^* had similar characteristics to those of the control group, with a similar percentage of SOX10^+^ cells in the corpus callosum (55.27% ± 5.7%; Fig. 4A), similar percentage of Ki67^+^ oligodendrocytes (18.21% ± 5.02%; Fig. 4B) and similar percentage of PDGF-Rα^+^ oligodendrocytes (1.99% ± 1.11%; Fig. 4C).

### Astrocytes

**Figure 5:**
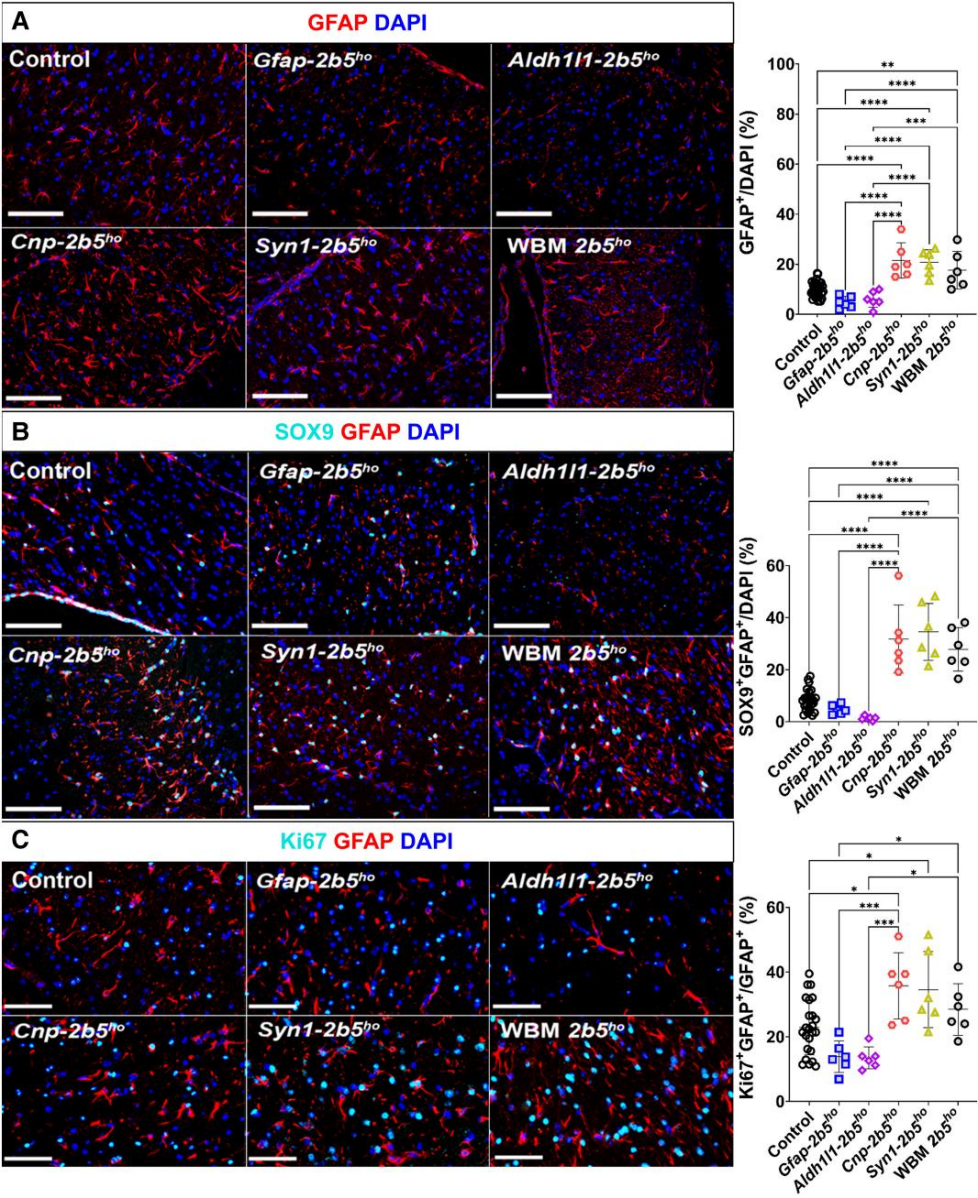
**Density of astrocytes.** All mice were 9 months old for this study. Sagittally cut brain sections through the corpus callosum from 24 conditional heterozygous mice (six per conditional line used as controls), six conditional homozygous mice per mutant line and six WBM-*2b5^ho^* mice were stained against: (**A**) GFAP; (**B**) SOX9 (cyan) and GFAP (red); and (**C**) Ki67 (cyan) and GFAP (red). All images were counterstained with DAPI. The corresponding quantification is shown on the *right* side of each panel. *P < 0.05, **P < 0.01, ***P < 0.001, ****P < 0.0001.

**Figure 6:**
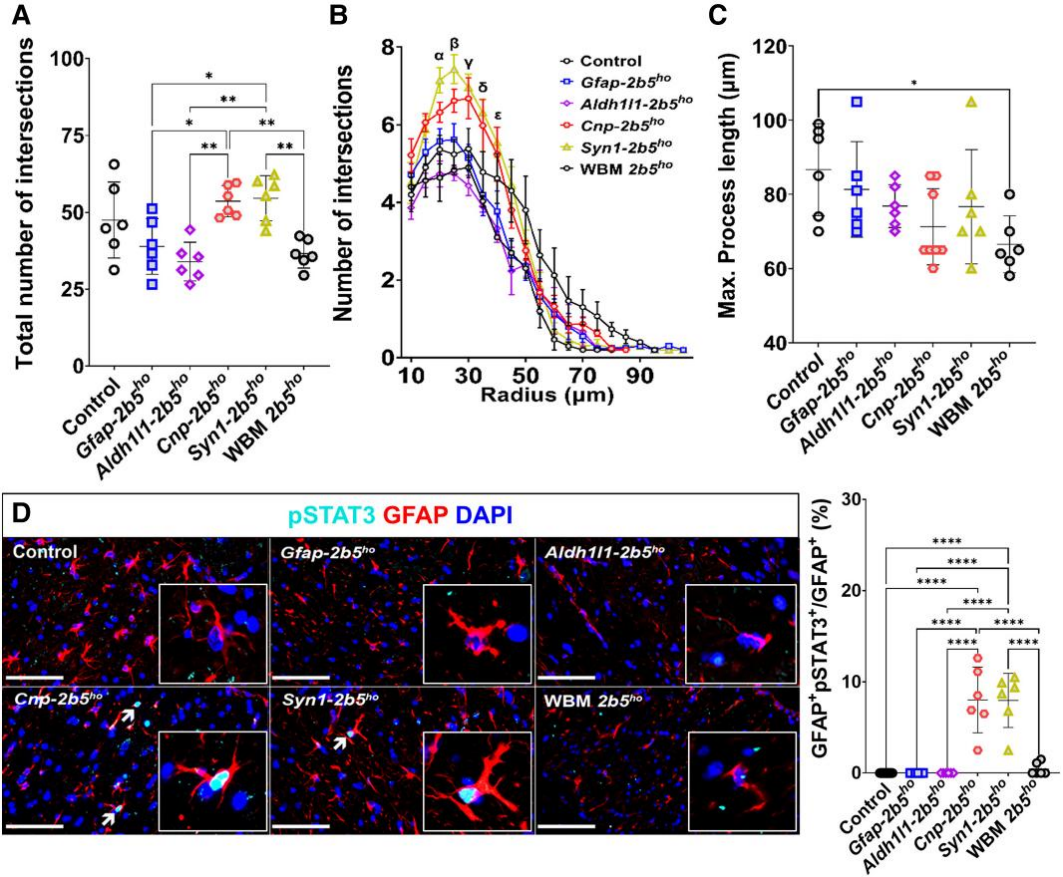
**Morphology and reactivity of astrocytes.** All mice were 9 months old for this study. Morphological analyses of astrocytes are based on five to seven randomly selected GFAP-stained astrocytes from the corpus callosum of six mice per line. (**A**–**C**) The total number of intersections (**A**), the number of intersections per radius (**B**) and the maximum process length (**C**) are displayed in the graphs. Each point represents the average for all astrocytes analysed in each mouse. (**D**) Representative images of immunofluorescence against pSTAT3 (cyan) and GFAP (red) counterstained with DAPI (blue). The arrows indicate the presence of nuclear pSTAT3, indicative of the initial stages of reactive astrogliosis. Details of the astrocytes are shown in the inset (magnification 6 × the original image). Scale bars: 100 μm. *P < 0.05, **P < 0.01, ****P < 0.0001.

**Figure 7:**
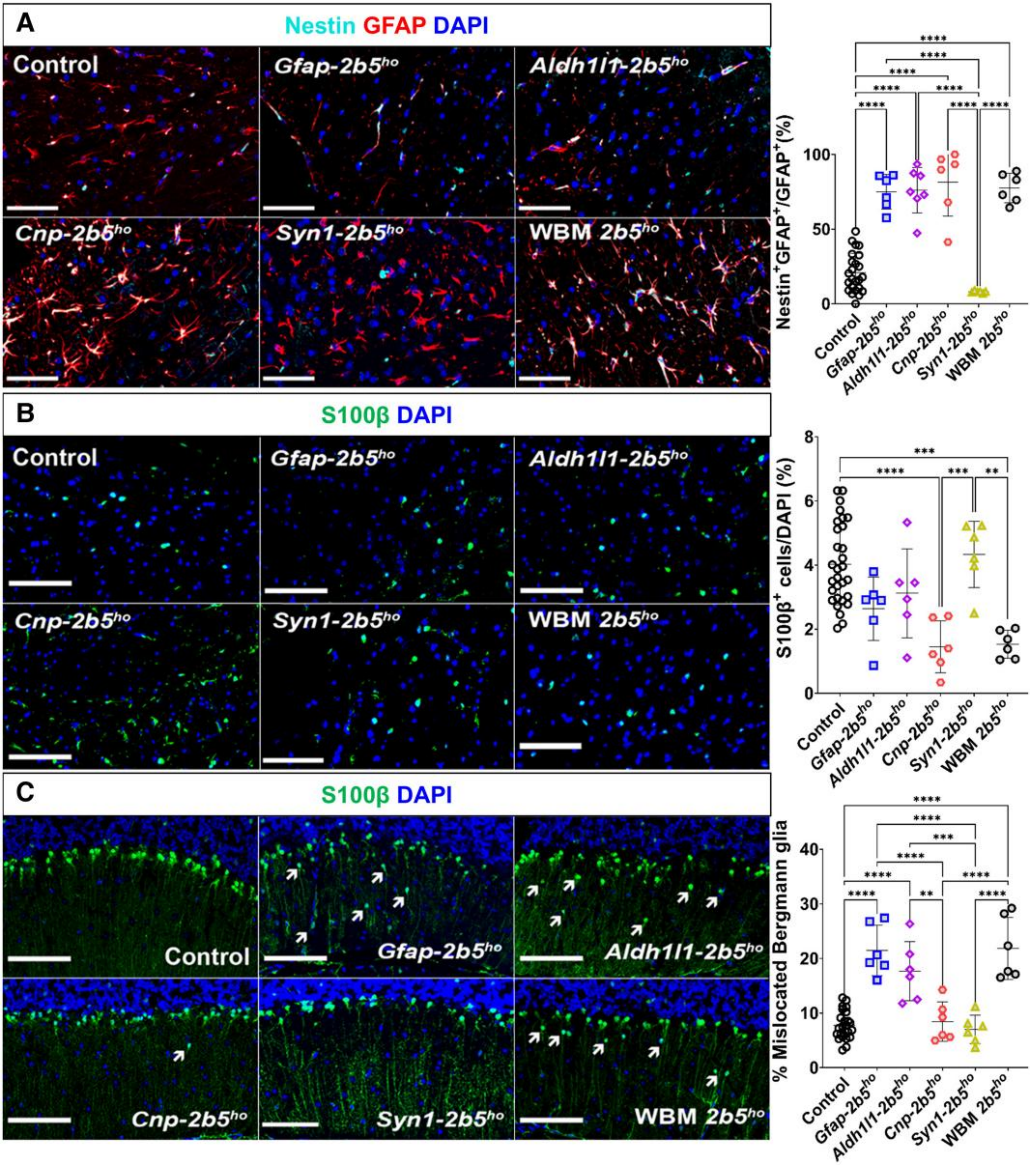
**Maturity of astrocytes.** All mice were 9 months old for this study. Sagittally cut brain sections from 24 conditional heterozygous mice (six per conditional line used as controls), six conditional homozygous mice per mutant line and six WBM-*2b5^ho^* mice were stained against: (**A**) Nestin (cyan) and GFAP (red) in the corpus callosum; and (**B**) S100β (green) in the corpus callosum. (**C**) S100β (green) was also analysed in the cerebellum to determine mislocalization of Bergmann glia. Arrows indicate those Bergmann glia cells that are mislocated. All images were counterstained with DAPI (blue). The corresponding quantification is shown on the *right* side of each panel. Scale bars: 100 μm. **P < 0.01, ***P < 0.001, ****P < 0.0001.

Within the corpus callosum, *Gfap-2b5^ho^* mice did not show significant differences in astrocyte density compared with control mice based on GFAP^+^ cells (4.83% ± 2.31% versus 9.98% ± 2.98%; Fig. 5A) and double SOX9^+^GFAP^+^ cells (4.64% ± 1.81% versus 8.31% ± 4.24%; Fig. 5B). *Gfap-2b5^ho^* mice showed a lower percentage of GFAP^+^ (4.83% ± 2.31% versus 17.63% ± 7.49%, *P* < 0.0001; Fig. 5A) and GFAP^+^SOX9^+^ astrocytes (4.64% ± 1.81% versus 27.81% ± 8.31%, *P* < 0.0001; Fig. 5B) than WBM-*2b5^ho^* mice. The *Gfap-2b5^ho^* line had a lower percentage of cycling astrocytes (Ki67^+^GFAP^+^; 13.83% ± 4.86% versus 28.45% ± 8.00%, *P* < 0.05) than WBM-*2b5^ho^* mice (Fig. 5C). Cell death was not detected in astrocytes of the *Gfap-2b5^ho^* mice (Supplementary Fig. 5A–F). Sholl analysis did not show any differences between white matter astrocytes in the *Gfap-2b5^ho^* mice and controls or WBM-*2b5^ho^* mice (Fig. 6A–C). Reactive astrogliosis, defined as presence of double pSTAT3^+^GFAP^+^ cells, was not detected in *Gfap-2b5^ho^*, controls or WBM-*2b5^ho^* mice (Fig. 6D). The percentage of immature astrocytes (GFAP^+^nestin^+^) in *Gfap-2b5^ho^* mice was higher than in controls (74.98% ± 11.66% versus 20.79% ± 13.08%, *P* < 0.0001) and similar to WBM-*2b5^ho^* mice (77.98% ± 11.66%) (Fig. 7A). The percentage of fully mature astrocytes, characterized by uniform perinuclear staining for S100β, of all cells in the corpus callosum, in *Gfap-2b5^ho^* mice (2.63% ± 0.99%) was in between controls (4.02% ± 1.26%) and WBM-*2b5^ho^* mice (1.53% ± 0.43%) (Fig. 7C). *Gfap-2b5^ho^* mice exhibited an increased number of mislocalized Bergmann glia in comparison to controls (21.48% ± 4.60% versus 7.75% ± 2.61%, *P* < 0.0001) and similar to WBM-*2b5^ho^* mice (Fig. 7C).

*Aldh1l1-2b5^ho^* mice had similar numbers of GFAP^+^ astrocytes in the corpus callosum to controls (6.00% ± 3.22% versus 9.98% ± 2.98%) and lower than WBM-*2b5^ho^* mice (17.63% ± 7.49%, *P* < 0.0001) (Fig. 5A). GFAP^+^SOX9^+^ quantification in the corpus callosum of *Aldh1l1-2b5^ho^* did not show differences from control mice (1.35% ± 0.77% versus 8.31% ± 4.24%) but showed lower cell numbers than in WBM-*2b5^ho^* mice (27.81% ± 8.31%, *P* < 0.0001) (Fig. 5B). The percentage of double Ki67^+^GFAP^+^ cells in the corpus callosum of Aldh1l1-*2b5^ho^* mice did not differ from controls (13.49% ± 3.39% versus 22.88% ± 8.44%) and was lower than in WBM-*2b5^ho^* mice (28.45% ± 8.00%, *P* < 0.05) (Fig. 5C). Apoptosis was undetectable (Supplementary Fig. 5A). Sholl analysis did not reveal morphological abnormalities (Fig. 6A–C). No evidence of reactive astrogliosis was detected in the corpus callosum of Aldh1l1-*2b5^ho^* mice (Fig. 6D). The percentage of immature astrocytes in the *Aldh1l1-2b5^ho^* mice was increased compared with controls (76.11% ± 15.24% versus 20.79% ± 13.08%, *P* < 0.0001) and comparable to WBM-*2b5^ho^* mice (77.98% ± 11.66%) (Fig. 7A). The percentage of mature S100β^+^ astrocytes in the corpus callosum of the *Adh1l1-2b5^ho^* mice (3.12% ± 1.39%) was in between controls (4.02% ± 1.26%) and WBM-*2b5^ho^* mice (1.53% ± 0.43%) (Fig. 7B). Bergmann glia translocation was higher in *Adh1l1-2b5^ho^* mice than in controls (17.67% ± 5.43% versus 7.75% ± 2.61%; *P* < 0.0001), similar to what was observed in *Gfap-2b5^ho^* (21.48% ± 4.60%) and WBM-*2b5^ho^* mice (21.84% ± 5.71%) (Fig. 7C).

Oligodendrocyte-specific *Cnp-2b5^ho^* mice showed a higher immunoreactivity for GFAP in the corpus callosum than controls (21.50% ± 7.06% versus 9.98% ± 2.98%, *P* < 0.0001) and astrocyte-specific mutant mice, and similar to WBM-*2b5^ho^* mice (17.63% ± 7.49%) (Fig. 5A). Likewise, the number of SOX9^+^GFAP^+^ astrocytes in the corpus callosum of *Cnp-2b5^ho^* mice was higher than in controls (31.78% ± 13.08% versus 8.31% ± 4.24%, *P* < 0.0001) and astrocyte-specific mutant mice and was comparable to the WBM-*2b5^ho^* mice (27.81% ± 8.31%) (Fig. 5B). *Cnp-2b5^ho^* mice showed increased numbers of cycling astrocytes in the corpus callosum compared with controls (35.75% ± 10.22% versus 22.88% ± 8.44%, *P* < 0.05) and astrocyte-specific mutant mice, and similar to WBM-*2b5^ho^* mice (28.45% ± 8.00%) (Fig. 5C). Apoptosis was not observed (Supplementary Fig. 5A). In the Sholl analysis, processes of *Cnp-2b5^ho^* astrocytes showed more intersections than *Gfap-2b5^ho^* mice (47.49 ± 12.35 versus 38.92 ± 9.05, *P* < 0.05), *Aldh1l1-2b5^ho^* mice (33.95 ± 6.30, *P* < *0.01*) and WBM-*2b5^ho^* mice (36.57 ± 4.69, *P* < 0.01) (Fig. 5A–C). Reactive astrogliosis, as indicated by pSTAT3 positivity, was detected in a few GFAP^+^ cells per slice of the corpus callosum of *Cnp-2b5^ho^* mice (Fig. 6D). *Cnp-2b5^ho^* GFAP^+^ astrocytes had an increased expression of nestin compared with controls (81.59 ± 22.71 versus 20.79 ± 13.08, *P* < 0.0001), but similar to astrocyte mutant lines and the WBM-*2b5^ho^* animals (Fig. 7A). S100β^+^ astrocytes in the corpus callosum of *Cnp-2b5^ho^* mice showed a cytoplasmic staining pattern, indicative of immature astrocytes or astrocytes responding to pathological conditions, which was not observed in any other line, including WBM-*2b5^ho^* mice (Fig. 7B). When exclusively quantifying the perinuclear S100β^+^ staining characteristic of mature astrocytes, the *Cnp-2b5^ho^* line was the only conditional mouse line with a lower number in the corpus callosum than control mice (1.45% ± 0.81% versus 4.02% ± 1.26%, *P* < 0.0001) and similar to the WMB-*2b5^ho^* mice (1.53% ± 0.43%) (Fig. 7B). Strikingly, Bergmann glia localization in the *Cnp-2b5^ho^* was normal (Fig. 7C).

White matter astrocytes in the *Syn1-2b5^ho^* mice showed increased density of GFAP^+^ astrocytes compared with controls (20.76% ± 5.03% versus 9.98% ± 2.98%, *P* < 0.0001) and similar to the numbers in *Cnp-2b5^ho^* (21.50% ± 7.06%) and WBM-*2b5^ho^* mice (17.63% ± 0.49%) (Fig. 5A). The density of SOX9^+^GFAP^+^ astrocytes in the corpus callosum of *Syn1-2b5^ho^* mice was higher than in controls (34.51% ± 10.93% versus 8.31% ± 4.24%, *P* < 0.0001) and similar to *Cnp-2b5^ho^* and WBM-*2b5^ho^* mice (Fig. 5B). *Syn1-2b5^ho^* mice showed increased numbers of proliferative astrocytes in the corpus callosum compared with controls (35.75% ± 10.22% versus 22.88% ± 8.44%, *P* < 0.05) and similar to *Cnp-2b5^ho^* and WBM-*2b5^ho^* mice (28.45% ± 8.00%) (Fig. 5C). *Syn1-2b5^ho^* astrocytes had a similar morphology to astrocytes in the *Cnp-2b5^ho^* line, which was confirmed by Sholl analysis (Fig. 6A). Thus, *Syn1-2b5^ho^* astrocytes showed a higher number of intersections than astrocytes in *Gfap-2b5^ho^* (54.60 ± 7.35 versus 38.92 ± 9.05, *P* < 0.05) and *Aldh1l1-2b5^ho^* (33.95 ± 6.30, *P* < 0.01) lines and WBM-*2b5^ho^* mice (36.57 ± 4.69, *P* < 0.01) (Fig. 6A–C). As in *Cnp-2b5^ho^* mice, *Syn1-2b5^ho^* mice showed a few reactive astrocytes per slice in the corpus callosum (Fig. 6D). However, the percentage of astrocytes from *Syn1-2b5^ho^* mice expressing nestin was the lowest of all lines (8.06% ± 0.70%; Fig. 7A). The number of mature astrocytes with a perinuclear S100β^+^ staining among all cells in the corpus callosum in the *Syn1-2b5^ho^* mice did not differ from that in controls (4.33% ± 1.04% versus 4.02% ± 1.26%; Fig. 7B). Bergmann glia location was normal in *Syn11-2b5^ho^* mice (Fig. 7C).

### ATF4 transcriptome

**Figure 8:**
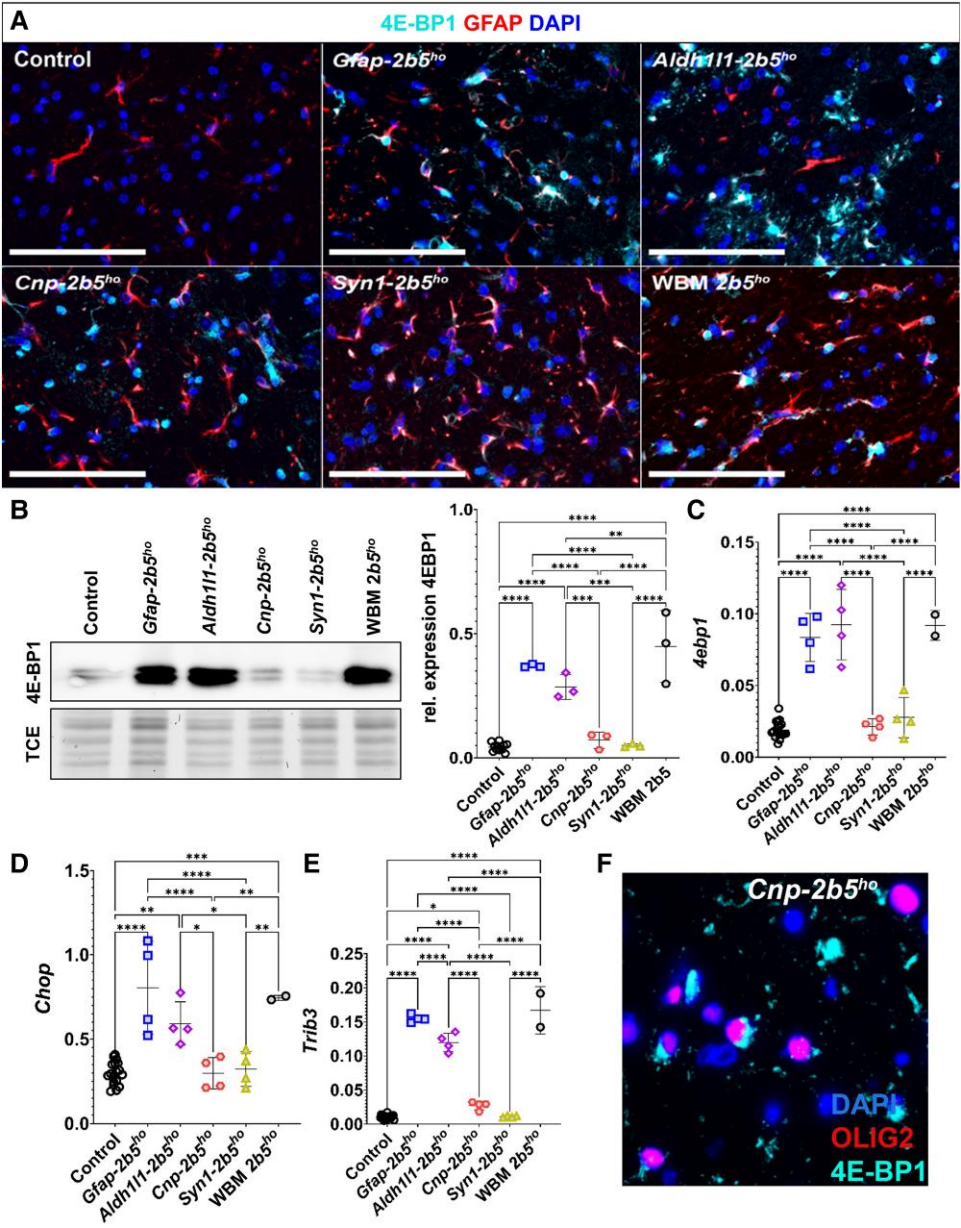
**ATF4 transcriptome.** (**A**) All mice were 9 months old for this study. Sagittally cut brain sections from 24 conditional heterozygous mice as controls, six conditional homozygous mice per mutant line and six WBM-*2b5^ho^* mice were stained against 4E-BP1 (cyan), GFAP (red) and DAPI (blue). The images are representative figures of the corpus callosum obtained after staining with the Cell Signaling 4E-BP1 antibody 9644S. All mutant lines showed some degree of 4E-BP1 staining in GFAP^+^ cells. The staining in the *Cnp-2b5^ho^* line also showed immunoreactivity in the perinuclear area of GFAP^−^ cells. (**B**–**D**) For this study, heterozygous and homozygous conditional mice were 9 months old, whereas wild-type (WT) and WBM-*2b5^ho^* mice were 6 months old. (**B**) Representative image of a western blot against 4E-BP1 using whole cerebellum lysates and the quantification performed on 12 control mice (10 conditional heterozygous and two WT), three conditional homozygous mutant mice per line and three WBM-*2b5^ho^* mice. TCE = 2,2,2-trichloroethanol staining. (**C**) Quantification of *4ebp1* mRNA in the cerebellum of 18 control mice (16 conditional heterozygous and two WT), four conditional homozygous animals per mutant line and two WBM-*2b5^ho^* mice. The same animals and samples as in **C** were used to quantify *Chop* mRNA (**D**) and *Trib3* mRNA (**E**). (**F**) Representative image of the staining against 4E-BP1 (cyan) and OLIG2 (red) in the corpus callosum of the *Cnp-2b5^ho^* line. The Atlas 4E-BP1 antibody HPA023501 also detected increased expression of 4E-BP1 in OLIG2 cells even when western blots and quantitative PCR analysis did not show it. Scale bars: 100 μm. *P < 0.05, **P < 0.01, ***P < 0.001, ****P < 0.0001.

Immunostaining against 4E-BP1 in the WBM-*2b5^ho^* animals showed expression in both astrocytes (Fig. 8A) and oligodendrocytes (Supplementary Fig. 6A and B). 4E-BP1 expression was not detected in the controls (Fig. 8A and Supplementary Fig. 6A and B). Western blot against 4E-BP1 in the cerebellum of the animals confirmed the differences between WBM-*2b5^ho^* mice and control mice (0.45 ± 0.14 versus 0.04 ± 0.01; *P* < 0.0001; Fig. 8B and Supplementary Fig. 7A). *Gfap-2b5^ho^* mice showed increased 4E-BP1 immunoreactivity that was detectable only in astrocytes (Fig. 8A), but not in oligodendrocytes (Supplementary Fig. 6A and B). *Gfap-2b5^ho^* mice had an increased expression of the ISR-related genes *4ebp1*, *Chop* and *Trib3* in comparison to controls and similar to WBM-*2b5^ho^* animals (Fig. 8C–E).

Comparable to the *Gfap-2b5^ho^*, *Aldh1l1-2b5^ho^* mice showed higher expression of 4E-BP1 in the cerebellum than controls (0.28 ± 0.05 versus 0.04 ± 0.01; *P* < 0.0001; Fig. 8B), and the staining indicated that this ISR protein is in astrocytes (Fig. 8A). However, GFAP expression in *Aldh1l1-2b5^ho^* mice was the lowest of all conditional lines, making the expression of 4E-BP1 more pronounced than GFAP expression (Fig. 8A). Co-staining of 4E-BP1 and OLIG2 showed expression of 4E-BP1 in some oligodendrocytes, although in very few (Supplementary Fig. 6A). As in the other astrocyte-specific line, *Aldh1l1-2b5^ho^* mice had increased expression of *4ebp1*, *Chop* and *Trib3* in the cerebellum (Fig. 8C–E).

The *Cnp-2b5^ho^* mice showed 4E-BP1 expression in some GFAP^+^ astrocytes (Fig. 8A), although 4E-BP1 expression was detected mainly with a (peri)nuclear pattern in OLIG2^+^ cells (Supplementary Fig. 6A and B). However, western blot analysis of the cerebellum did not show increased 4E-BP1 expression (Fig. 8B and Supplementary Fig. 7A), and gene expression levels of *4ebp1* and *Chop* were similar to those of controls (Fig. 8C and D), while *Trib3* expression was higher than in controls (0.02 ± 0.006 versus 0.01 ± 0.003; *P* < 0.05) but much lower than in WBM-*2b5^ho^* (0.16 ± 0.03) and astrocyte-specific mutant mice (0.15 ± 0.005 in *Gfap-2b5^ho^*, 0.12 ± 0.01 in *Aldh1l1-2b5^ho^*) (Fig. 8E). Given the contradictory results between 4E-BP1 immunostaining, western blot and qPCR, a second antibody was used to exclude non-specific binding in the immunostaining. This second antibody also showed 4E-BP1 immunoreactivity in *Cnp-2b5^ho^* mice being especially intense in oligodendrocytes (Fig. 8F and Supplementary Fig. 6B), but it did not show specific detection of 4E-BP1 by western blot (Supplementary Fig. 7B). The reason for the immunoreactivity observed in oligodendrocytes could not be determined.

*Syn1-2b5^ho^* mice showed a 4E-BP1 staining similar to the WBM-*2b5^ho^* animals, with 4E-BP1 present in both astrocytes (Fig. 8A) and oligodendrocytes (Supplementary Fig. 6A). As for the *Cnp-2b5^ho^* line, western blot against 4E-BP1 and gene expression levels of *4ebp1*, *Chop* and *Trib3* were similar to those of controls (Fig. 8C–E).

## Discussion

Selective vulnerability of specific brain cell types in VWM has been a subject of extensive research.^8,11,13,16,17,30,31^ Previous histopathological analyses of brain tissue from VWM patients and VWM mouse models suggested that VWM primarily affects astrocytes and oligodendrocytes, while neurons are relatively preserved.^11,12,14-16,32^ Co-culture studies suggested that astrocyte disease is primary, with oligodendrocyte dysfunction and minor neuronal abnormalities being secondary to astrocyte disease.^14-16^ By using conditional *Eif2b5* mouse models, in the present study we delineate the contribution of astrocytes, oligodendrocytes and neurons to the development and progression of VWM.

Our results indicate that astrocyte pathology has a minimal impact on the development of gait ataxia in VWM, with *Gfap*- and *Aldh1l1-2b5^ho^* mice developing a phenotype characterized by kyphosis, never reported in any previous VWM model and also not observed in VWM patients. Clearly, astrocytes are important for the development of the intramyelinic vacuoles characteristic of VWM.^16^ Despite the supporting role of astrocytes, oligodendrocytes do not exhibit any neuropathological abnormality in astrocyte-specific *Eif2b5* mutant mice. Astrocytes, in contrast, are severely affected: they are scarce, with few processes, and display increased expression of the immature marker nestin, which has been reported in VWM patients^11^ and mouse models.^16^ Bergmann glia mislocalization, another previously described marker of VWM,^17^ is present in these lines. Also, the constitutively increased levels of ATF4-driven factors in astrocytes, as observed in VWM patient brains^8,9^ and mouse models,^8,18,24,29^ are replicated in both astrocyte-specific mutant lines with an increased expression of 4E-BP1 in astrocytes.^8^ Regarding the relationship between pathology and the (lack of) clinical phenotype, it is known that intramyelinic vacuolization can be present without causing motor dysfunction or other clinical manifestations. An example is megalencephalic leukoencephalopathy with subcortical cysts, in which extensive myelin vacuolization is present without neurological dysfunction in the first years of life.^33^ Therefore, although intramyelinic vacuolization can be a marker of underlying white matter pathology, its presence alone is not necessarily correlated with motor dysfunction. We also conclude that Bergmann glia mislocalization is not correlated with clinical ataxia.

Clearly, oligodendrocytes play a primary role in the development of the gait ataxia. The phenotype of *Cnp-2b5^ho^* mice is most comparable to that of the WBM-*2b5^ho^* mice, with a CAS and CatWalk performance matching that of WBM-*2b5^ho^* mice and previously described VWM mouse models.^24^  *Cnp-2b5^ho^* mice do not exhibit intramyelinic vacuoles, supporting the role of mutant astrocytes in their development, but the number of myelinated axons is reduced, as previously reported in the WBM-*2b5^ho^* mice.^15^  *Cnp-2b5^ho^* mice have a decreased density of oligodendrocytes in the corpus callosum. They have an increased proportion of oligodendrocyte precursor cells, as described in VWM patients,^11^ and a strikingly high percentage of oligodendrocytes is in the active cell cycle. In this mouse line, white matter astrocytes are also affected, displaying a cytoplasmic pattern of S100β staining that suggests stress.^34^ The density of immature astrocytes is increased, a characteristic previously described in VWM patients^11^ and WBM-*2b5^ho^* mice.^16^ Interestingly, the expression of the ATF4 transcriptome is completely normal in the brains of *Cnp-2b5^ho^* animals. Regarding the relationship between pathology and the clinical phenotype, oligodendrocyte dysfunction and lack of myelinated axons might contribute to the clinical phenotype. Although the mechanism is not clear, we would like to point out that ataxia is a central clinical feature of hypomyelinating disorders.^35^ The normal Bergmann glia localization in this line confirms a disconnection between Bergmann glia mislocalization and ataxia.

The neuron-specific *Eif2b5* mutant line exhibits a very mild phenotype, in line with a minimal impact of neurons on disease development in VWM. They have slight pelvic tilt but no other major VWM features. Myelin looks normal, without intramyelinic vacuoles and with normal levels of myelin proteins. *Syn1-2b5^ho^* animals do not exhibit changes in the number of oligodendrocytes, cell cycle state or maturation. There is an increased density of astrocytes with increased ramifications and presence of reactive astrogliosis, indicating an astrocytic response. Unlike other VWM models, astrocytes from *Syn1-2b5^ho^* mice do not overexpress nestin. In *Syn1-2b5^ho^* animals, Bergmann glia localization is normal, and there is no enhanced ATF4-related gene expression.

Our study reveals that VWM pathophysiology is more complex than previously thought. Earlier research suggested astrocytes to be key players^16^ and therefore therapeutic targets for VWM.^36^ However, the present study suggests that mutant oligodendrocytes are also crucial drivers of the disease. Gait ataxia is the central clinical manifestation in VWM patients^37^ and VWM mice.^16^ Our oligodendrocyte-specific *Eif2b5* mutant mouse line exhibits this phenotype along with several neuropathological and molecular characteristics observed in VWM models. The reduction in oligodendrocyte density (SOX10^+^ cells) with a notable increase in both cycling (Ki67^+^SOX10^+^) and immature (PDGF-Rα^+^SOX10^+^) oligodendrocytes could be a relevant neuropathological feature in the development of VWM. The increase in Ki67 expression has been observed in oligodendrocyte precursor cells in VWM patients^11^ and other models with myelination defects.^38,39^ Ki67 is a proliferation marker that is degraded in the G0 phase and accumulates during the S, G2 and M phases of the cell cycle.^40^ Oligodendrocyte differentiation and maturation require exiting the cell cycle and activating a set of myelin-related genes.^41^ The presence of Ki67 in oligodendrocytes without an increase in cell density, together with the homogeneous pattern of nuclear staining, suggests that these cells might be experiencing cell cycle arrest.^42,43^ Herrero *et al*.^30^ found impaired differentiation capacity in oligodendrocytes isolated from *Eif2b5* mutant mice and suggested that their defective maturation could be a consequence of the extensive mitochondrial dysfunction observed in these cells. Mitochondrial dysfunction, cell cycle arrest and defective maturation could all be mechanisms driving the disease in oligodendrocytes. Importantly, the mutant oligodendrocytes also affect astrocytes. In *Gfap*− and *Aldh1l1-2b5^ho^* mice the oligodendrocytes are not affected, whereas *Cnp-2b5^ho^* mice display abnormalities not only in oligodendrocytes, but also in astrocytes.

With the present results, the role of astrocytes in disease development in VWM has become less clear. Although astrocyte-specific *Eif2b5* mutant mice do not develop the clinical VWM phenotype, they do exhibit several neuropathological key features associated with VWM that are absent in other conditional lines. These features include intramyelinic vacuoles, indicating the crucial role of astrocytes in maintaining brain water homeostasis; Bergmann glia mislocalization, which must result from a cell-autonomous effect of *Eif2b5* mutations in astrocytes; and the increased expression of ATF4-related genes. All these classical markers of VWM appear to be related directly to mutant astrocytes, although their relationship with clinical manifestations is not clear.

The study presents some limitations. The *Eif2b5* mouse model replicates Cree leukoencephalopathy caused by a homozygous p.Arg195His variant in *EIF2B5*. Patients with Cree leukoencephalopathy typically have a disease onset in the second half of the first year of life and are terminal before the age of 2 years.^44^ That is exactly what is seen in the homozygous p.Arg191His mutant *Eif2b5* mouse model. However, although working with a mouse model truly representative of human disease, the present study provides data at only one time point, 9 months of age, for all conditional *Eif2b5* mutant lines, without insight into the time line and sequence of events. Other limitations are intrinsic to the use of conditional models. First, astrocytes are highly responsive to their surrounding cells and environment, increasing GFAP expression and replication as a response to insults; being surrounded by a healthy environment might be responsible for the low number of GFAP^+^ and SOX9^+^ cells found in the astrocyte-conditional lines in comparison with the oligodendrocyte-conditional and WBM-*2b5^ho^* mice. Sholl analysis showed that neuron- and oligodendrocyte-conditional models had the most complex astrocytes, and pSTAT3^+^ nuclei indicate a reactive phenotype. The study results indicate that not only cellular pathology, but most of all complex intercellular interactions underlie the development of VWM. It is therefore crucial to study other cell types further, especially microglia. In the *Aldh1l1-2b5^ho^* mice, in which the mutation is induced by tamoxifen injections,^22^ the timing of the mutations might not have aligned with critical windows of oligodendrocyte development. However, there were no essential differences between the *Aldh1l1-2b5^ho^* mice and the *Gfap*-*2b5^ho^* mice, negating this possibility.

An important limitation of the study is the lack of correlation between the stains for the ATF4-regulated gene *4E-BP1* and both qPCR and western blot data. Immunostaining shows increased expression of 4E-BP1 in the corpus callosum of *Gfap-2b5^ho^*, *Aldh1l1-2b5^ho^*, *Cnp-2b5^ho^* and *Syn1-2b5^ho^* mice. However, qPCR and western blot data show enhanced expression of ATF4-regulated genes only in astrocyte-specific and WBM lines. We performed stains with several antibodies with similar results. We cannot conclude whether there is a cell-specific mechanism involved in the immunoreactivity observed in all lines or whether it is simply attributable to non-specific binding of 4E-BP1 antibodies. In any case, in our experimental conditions the astrocyte-specific *Eif2b5* and WBM-*2b5^ho^* mutant mice showed the highest expression of the ATF4-regulated transcriptome, suggesting that the effects of *Eif2b5* mutation on the ATF4 transcriptome mostly affect astrocytes. This could be explained by the high sensitivity of astrocytes to the negative effect of eIF2B partial loss of function.^45^ We cannot exclude the possibility that oligodendrocyte- and neuron-specific *Eif2b5* mutant mice suffer an increased expression of the ATF4-regulated transcriptome at a developmental stage that was not captured by our study design, although in our previous studies covering more ages we did not find evidence for that.^8^

## Conclusion

In conclusion, our findings in astrocyte-, oligodendrocyte- and neuron-specific mutant lines reveal a complex interaction among various brain cell types in VWM. Contrary to the initial premise of astrocytes driving VWM pathology, our findings underscore a paradigm shift towards a more intricate relationship, whereby astrocytes appear to be responding to disease in oligodendrocytes rather than being the initiators. The conclusion that oligodendrocytes drive the disease would be an oversimplification, because it is clear that the astrocyte-specific line displays several features typical of VWM, not observed in the oligodendrocyte-specific line. This study challenges the view of a singular cell population driving VWM pathology, but it is safe to conclude that oligodendrocytes and astrocytes, together the macroglia, play much more important roles than neurons in development of the disease. The contribution of other cell types, especially microglia, still needs to be investigated. Unravelling the intricate relationships between different cell types is pivotal not only for a deeper comprehension of VWM pathogenesis but also for the development of precise and targeted therapeutic strategies aimed at mitigating its impact.

## Supplementary Material

awaf171_Supplementary_Data

## Data Availability

The data that support the findings of this study are available from the corresponding author, upon reasonable request.
