# Supplementary material for: Cell-specific Eif2b5 mutant mice: novel insights into roles of macroglia in vanishing white matter
Source: Brain. 2025 May 6;148(11):4112–26. doi: 10.1093/brain/awaf171 (PMC12588717; doi:10.1093/brain/awaf171)
Supplement: awaf171_Supplementary_Data [file awaf171_supplementary_data.zip › brain-2024-02266-File010.pdf]

Supplementary Table 1 Comparison of CatWalk parameters between control mice and each of the conditional homozygous group and WBM-*2b5*^{ho}.

		Control	*Gfap-2b5*^{ho}	*Aldh1l1-2b5*^{ho}	*Cnp-2b5*^{ho}	*Syn1-2b5*^{ho}	WBM 2b5
**Run Characterization**	Duration (s) - all	2,20	2,21	2,88	6,07 ***	2,36	4,38 ***
	Cadence (steps/s) - all	13,38	14,34	15,59	7,22 ***	12,95	8,49 ***
	Average speed (cm/s) - all	25,84	26,76	25,97	9,66 ***	23,73	12,55 ***
	Number of steps - all	63,01	63,75	82,57	107,35 ***	67,30	101,56 **
**Interlimb coordination**	Step Sequence - all	12,85	13,00	14,19	21,00 ***	12,95	20,94 ***
	Support single (%) - all	1,55	3,29 **	4,51 ***	4,07 ***	1,65	5,14 ***
	Support diagonal (%) - all	62,22	68,16	60,38	38,13 ***	59,68	41,95 ***
	Support girdle (%) - all	1,75	1,21	4,49 ***	2,86 ***	3,47 **	3,22 ***
	Support lateral (%) - all	0,90	1,20	2,77 ***	3,46 ***	1,16	4,40 ***
	Support three (%) - all	27,24	21,64	21,89	35,12 **	25,74	35,95 **
	Stride length FP (cm) - FP	7,35	7,50	6,96 **	5,05 ***	6,97	5,46 ***
	Stride length HP (cm) - HP	7,21	7,11	6,39	5,09 ***	6,73	5,47 ***
**Temporal**	Initial dual stance (s) - FP	0,03	0,02	0,03	0,10 ***	0,03	0,06 ***
	Initial dual stance (s) - HP	0,04	0,03	0,03	0,12 ***	0,04	0,08 ***
	Stand (s) - FP	0,18	0,17	0,17	0,38 ***	0,18	0,29 ***
	Stand (s) - HP	0,18	0,17	0,19	0,42 ***	0,20	0,29 ***
	Swing (s) - FP	0,12	0,13	0,13	0,18 ***	0,12	0,17 ***
	Swing (s) - HP	0,11	0,11	0,11	0,19 ***	0,12	0,18 ***
	Step cycle (s) - FP	0,30	0,30	0,28	0,56 ***	0,30	0,46 ***
	Step cycle (s) - HP	0,30	0,28	0,26	0,59 ***	0,30	0,48 ***
	Terminal dual stance (s) - FP	0,03	0,02 *	0,05	0,10 ***	0,03	0,06 ***
	Terminal dual stance (s) - HP	0,04	0,03	0,07	0,14 ***	0,05	0,08 ***
	Single stance (s) - FP	0,12	0,12	0,10 ***	0,18 ***	0,11	0,17 ***
	Single stance (s) - HP	0,11	0,11	0,09 **	0,15 ***	0,11	0,15 ***
**Kinetic**	Body speed (cm/s) - FP	26,08	27,37	26,86	10,38 ***	24,62	12,34 ***
	Body speed (cm/s) - HP	25,50	26,95	26,62	10,13 ***	23,83	12,10 ***
	Swing speed (cm/s) - FP	64,57	64,34	66,84	33,14 ***	64,12	35,68 ***
	Swing speed (cm/s) - HP	67,74	64,13	63,47	36,07 ***	63,60	38,04 ***
	Stand index - FP	-5,78	-6,49	-8,08	-3,73 ***	-5,58	-3,70 ***
	Stand index - HP	-9,22	-10,50	-10,85	-5,01 ***	-9,09	-6,23 ***
	Body speed variation (cm/s) - FP	18,11	16,87	38,99 **	33,65 ***	26,42 **	21,26 **
	Body speed variation (cm/s) - HP	17,52	17,36	39,77 **	33,29 ***	25,73 **	21,28 **

*$p<0.05$; **$p<0.01$; ***$p<0.001$; ****$p<0.0001$.

Supplementary Table 2 Comparison of CatWalk parameters between WBM-*2b5ho*mice and each of the conditional homozygous group and control mice.

		Control		*Gfap-2b5*^{ho}		*Aldh1l1-2b5*^{ho}		*Cnp-2b5*^{ho}		*Syn1-2b5*^{ho}		WBM *2b5*
**Run Characterization**	Duration (s) - all	2,20	***	2,21	***	2,88	***	6,07	*	2,36	***	4,38
	Cadence (steps/s) - all	13,38	***	14,34	***	15,59	***	7,22	*	12,95	**	8,49
	Average speed (cm/s) - all	25,84	***	26,76	***	25,97	***	9,66	*	23,73	***	12,55
	Number of steps - all	63,01	**	63,75	***	82,57		107,35		67,30	***	101,56
**Interlimb coordination**	Step Sequence - all	12,85	***	13,00	**	14,19	*	21,00		12,95	***	20,94
	Support single (%) - all	1,55	***	3,29	**	4,51		4,07		1,65	***	5,14
	Support diagonal (%) - all	62,22	***	68,16	***	60,38	**	38,13		59,68	**	41,95
	Support girdle (%) - all	1,75	***	1,21	**	4,49		2,86		3,47		3,22
	Support lateral (%) - all	0,90	***	1,20	***	2,77		3,46		1,16	***	4,40
	Support three (%) - all	27,24	**	21,64	***	21,89	***	35,12		25,74	**	35,95
	Stride length FP (cm) - FP	7,35	***	7,50	***	6,96	**	5,05		6,97	**	5,46
	Stride length HP (cm) - HP	7,21	***	7,11	***	6,39		5,09		6,73	**	5,47
**Temporal**	Initial dual stance (s) - FP	0,03	***	0,02	***	0,03	***	0,10		0,03	***	0,06
	Initial dual stance (s) - HP	0,04	***	0,03	***	0,03	***	0,12		0,04	**	0,08
	Stand (s) - FP	0,18	***	0,17	***	0,17	**	0,38	*	0,18	**	0,29
	Stand (s) - HP	0,18	***	0,17	***	0,19	*	0,42	*	0,20	*	0,29
	Swing (s) - FP	0,12	***	0,13	***	0,13	***	0,18		0,12	*	0,17
	Swing (s) - HP	0,11	***	0,11	***	0,11	***	0,19		0,12	***	0,18
	Step cycle (s) - FP	0,30	***	0,30	**	0,28	*	0,56		0,30	***	0,46
	Step cycle (s) - HP	0,30	***	0,28	***	0,26	***	0,59		0,30	***	0,48
	Terminal dual stance (s) - FP	0,03	***	0,02	***	0,05		0,10		0,03	**	0,06
	Terminal dual stance (s) - HP	0,04	***	0,03	***	0,07		0,14		0,05	***	0,08
	Single stance (s) - FP	0,12	***	0,12	**	0,10	**	0,18		0,11	***	0,17
	Single stance (s) - HP	0,11	***	0,11	***	0,09	***	0,15		0,11	***	0,15
**Kinetic**	Body speed (cm/s) - FP	26,08	***	27,37	***	26,86	***	10,38		24,62	***	12,34
	Body speed (cm/s) - HP	25,50	***	26,95	***	26,62	***	10,13		23,83	***	12,10
	Swing speed (cm/s) - FP	64,57	***	64,34	***	66,84	***	33,14		64,12	***	35,68
	Swing speed (cm/s) - HP	67,74	***	64,13	***	63,47	***	36,07		63,60	***	38,04
	Stand index - FP	-5,78	***	-6,49	***	-8,08	***	-3,73		-5,58	***	-3,70
	Stand index - HP	-9,22	***	-10,50	***	-10,85	**	-5,01		-9,09	**	-6,23
	Body speed variation (cm/s) - FP	18,11	***	16,87	**	38,99	**	33,65		26,42		21,26
	Body speed variation (cm/s) - HP	17,52	***	17,36	**	39,77	**	33,29		25,73		21,28

*$p<0.05$; **$p<0.01$; ***$p<0.001$; ****$p<0.0001$.

Supplementary Table 3 Gene names and sequences of all primers used in the qPCR analyses.

Primer sequences used for qPCR analysis of specific genes.		
Gene name	Forward	Reverse
Chop	CTGGTATGAGGATCTGCAGG	TTGATTCTTCCTCTTCGTTTCC
Hprt	GTTGGGCTTACCTCACTGCT	TAATCACGACGCTGGGACTG
Mbp	GGACCCAAGATGAAAACCCAGTA	GGGATGGAGGTGGTGTTCG
Plp	CTGTGGATGTGGACATGAAGC	AGAAAGAGGCAGTTCCATAGATGA
Trib3	TGTCTTCAGCAACTGTGAGAGGACGAAG	GTAGGATGGCCGGGAGCTGAGTATC
4ebp1	CACGCTCTTCAGCACCACC	CCACAGGTGAGTTCCGACA

Supplementary Table 4 Table showing the primary antibodies used and the information about these antibodies.

Target	Type of marker	Manufacturer	Reference	Technique	Dilution
4E-BP1	Integrated stress response (cytoplasmic)	Cell Signaling	9644	IF	1/400
4E-BP1	Integrated stress response (cytoplasmic)	Atlas	HPA023501	IF	1/50
Caspase-3	Apoptosis (nuclear)	Cell Signaling	9661	IHC	1/100
CyclinD1	Proliferation (nuclear)	Thermo Scientific	MA5-16356	IHC	1/250
GFAP	Astrocytes (cytoplasmic)	Sigma Aldrich	AB5541	IF	1/1000
Ki67	Cell cycle (nuclear)	Thermo Scientific	RM-9106-S	IF	1/100
MBP	Mature myelin	Millipore	MAB387	WB	1/1000
Nestin	Immature astrocytes (cytoplasmic)	BD Bioscience	611658	IF	1/500
OLIG2	Oligodendrocytes (nuclear)	Cell Marque	387M	IHC	1/100
OLIG2	Oligodendrocytes (nuclear)	Sigma Aldrich	AB9610	IHC	1/400
Pan Neuronal	Neuron (all parts of the neuron)	Sigma Aldrich	MAB2300	IF	1/100
PDGF-Rα	Oligodendrocyte precursor cells (surface)	Cell Signaling	3174	IF	1/500
PLP/DM20	Mature myelin	Bio-Rad	MCA839G	WB	1/5000
pSTAT3	Reactive astroglyosis (nuclear)	Abcam	ab76315	IF	1/100
S100β	Mature astrocytes (perinuclear)	Proteintech	15146-1-AP	IF	1/1000
SOX10	Oligodendrocytes (nuclear)	Cell Marque	383R	IHC	1/100
SOX9	Astrocytes (nuclear)	Cell Signaling	82630	IHC	1/400

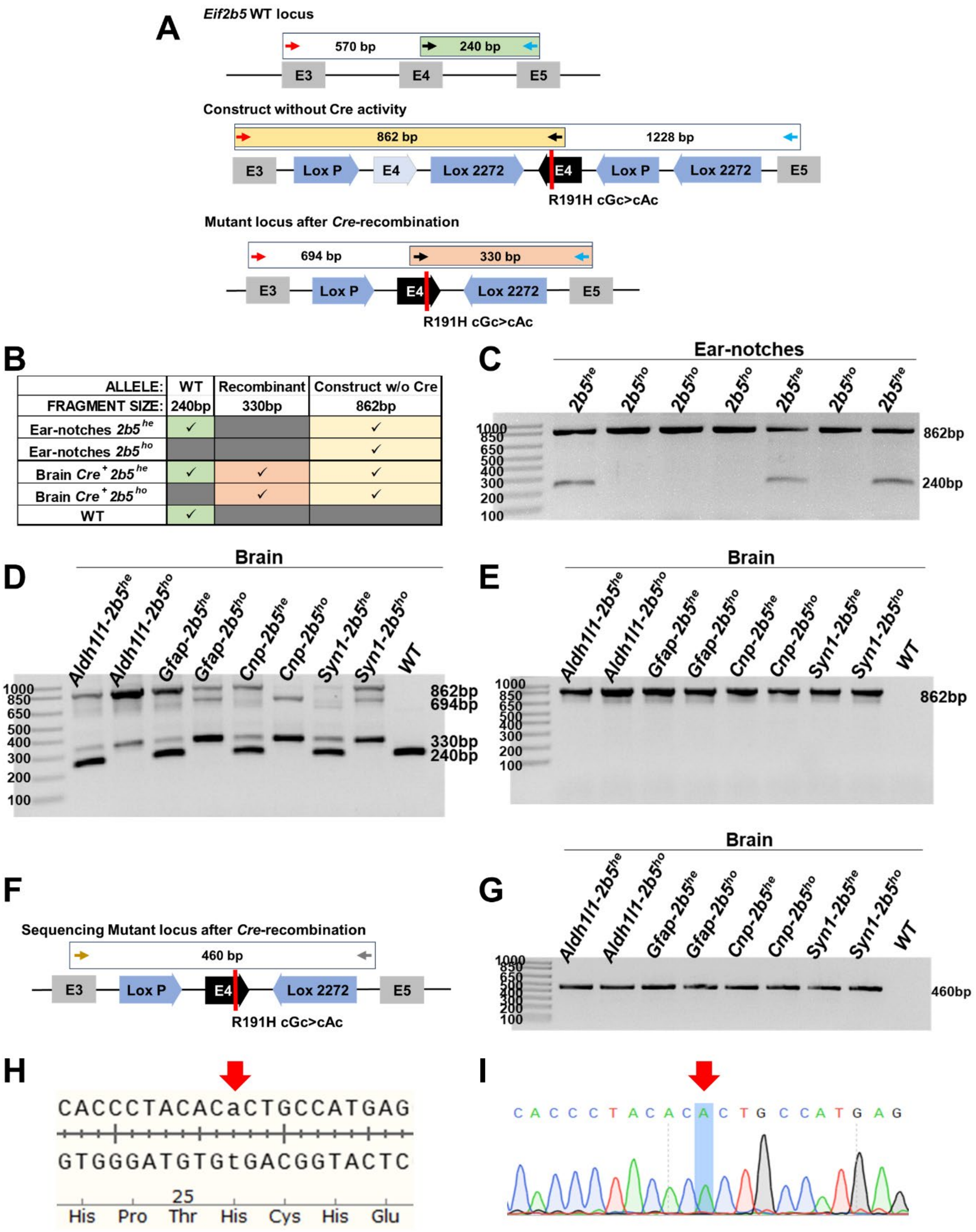

Supplementary Figure 1 Validation of the conditional *Eif2b5* mouse lines. (A) Diagram of the wildtype allele (top), construct without Cre activity (middle), and mutant locus after Cre-recombination (bottom) showing the location of the primers used for the genotyping and the detection of unspecific recombination. We used a PCR with three different primers to be able to distinguish all possible genotypes and to detect unspecific recombination in one single reaction. The colored amplicon is the predominant product of the PCR in each case. PCR was performed using the following primers AAGTGGTGCCATCCTACGTC (red); CTACACGCTGCCATGAGGAC (black); and GAATCTCCACTCCGTCTCCA (blue). In the contruct without Cre activity, Exon 4 (light blue) was modified using synonymous codon changes, as a result, the black primer does not bind it. **(B)** Table with the expected results in each genotype and type of sample. **(C)** Results obtained in a genotyping reaction using ear-notches. *Eif2b5* heterozygous mice have the 240 bp amplicon from the wildtype allele and the 862 bp amplicon from the construct. Homozygous *Eif2b5* mice have only the construct. Our breeding protocol did not produce wildtype animals for the *Eif2b5* allele. No unspecific recombination (330 bp amplicon) was not detected in the ear-notches. Ear-notches from all mice were

collected and lysed overnight at 55 °C in 0.1 mg/mL proteinase K. **(D)** when using the same PCR for analyzing gDNA from brain the 330 bp amplicon (mutant locus after Cre-recombination) is present in all samples. Only *Eif2b5* heterozygous mice have the 240 bp amplicon from the wildtype allele. The 862 bp amplicon produced very variable results due to the competition among the different amplicons during the PCR and the less efficient amplification of larger amplicons. **(E)** To clarify that all animals presented the 862 bp amplicon a PCR using only the red and black primers was performed, showing that all lines (except wildtype) have the construct. **(F)** To confirm the presence of the correct mutation a new PCR was performed in all lines. The golden primer binds exon 3 and the grey primer binds an artificial spacer region of the construct making sure that only the recombined sequences are amplified. **(G)** Results of the PCR for the sequencing of the mutant locus after Cre-recombination. **(H)** Expected sequence after recombination. **(I)** Results of the sequencing. PCR products were cleaned of free dNTPs by using ExoProStar (Illustra) and then sequenced using BigDye V3.1 (Applied Biosystems). All conditional lines were analyzed, and the correct mutation was confirmed in all of them.

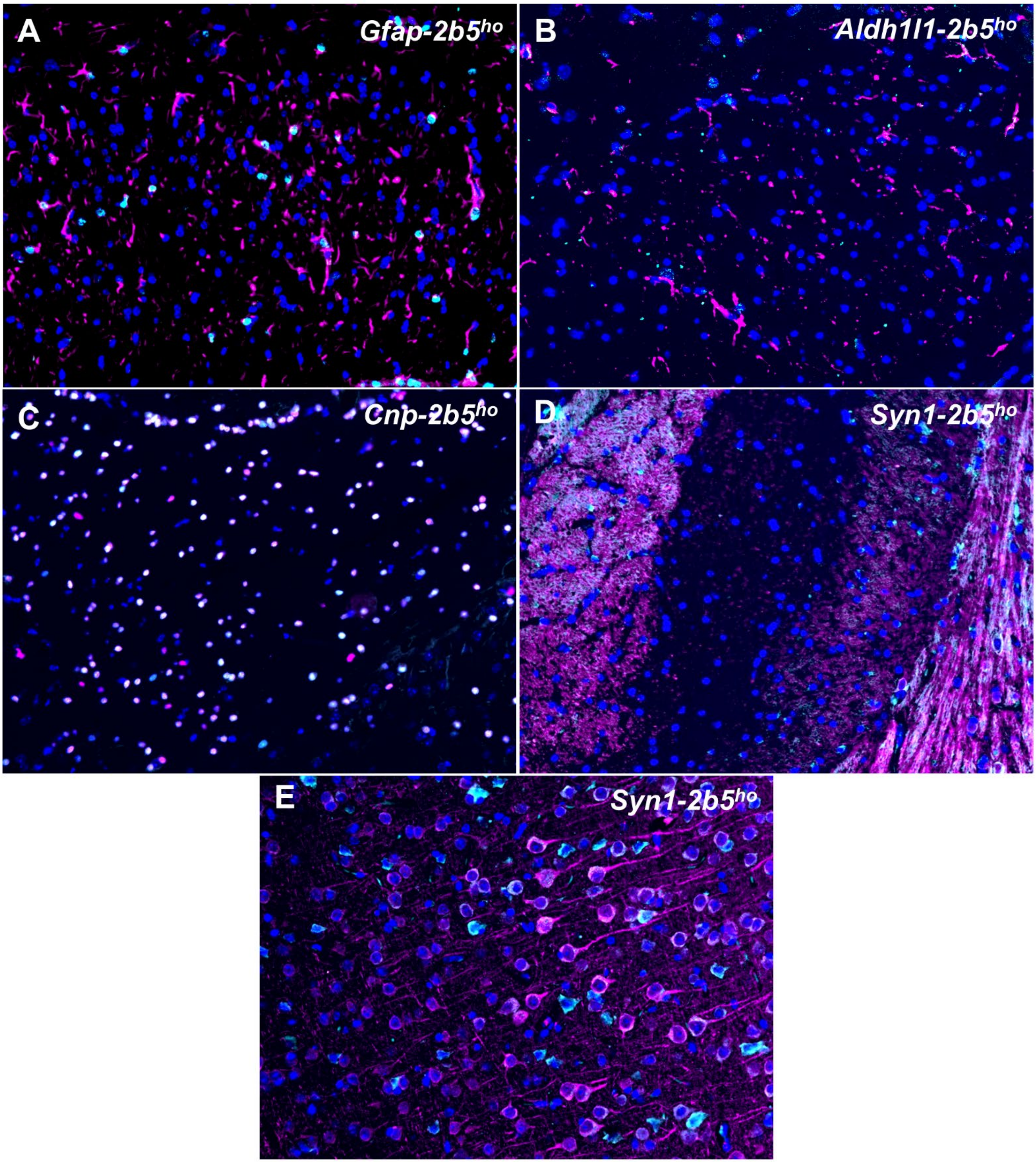

Supplementary Figure 2 Specificity of the Cre expression. At 9 months of age, sagittally cut brain sections from all *Eif2b5* conditional lines were stained against Cre recombinase (cyan). It is important to note that the expression of Cre at this time point is not indicative of the expression levels during the development of the animals and should only be considered as indicative of the specificity of its expression in the different cell types. Pictures of the splenium of the corpus callosum are shown in A-D, while a picture of the cortex of the *Syn1-2b5^{ho}* mouse is shown in D. **(A)** *Gfap-2b5^{ho}* mice showed expression of Cre (cyan) in GFAP^{+} cells (magenta) . **(B)** *Aldh1l1-2b5^{ho}* mice showed very low levels of Cre (cyan) at 9 months of age, which is also accompanied by very low levels of GFAP (magenta) and fits with the low number of astrocytic markers found in the white matter of this line (Figure 5). **(C)** *Cnp-2b5^{ho}* mice showed intense expression of Cre (cyan) in almost all OLIG2^{+} (magenta) cells. **(D)** Syn1-2b5ho mice showed no expression of Cre (cyan) in the corpus callosum, while only some positivity was detected in combination with a pan-neuronal marker (magenta). **(E)** To confirm the specificity of the Cre expression in the Syn1-2b5 line a picture of the cortex is shown to illustrate the positivity for Cre (cyan) in some cells stained with a pan-neuronal marker (magenta).

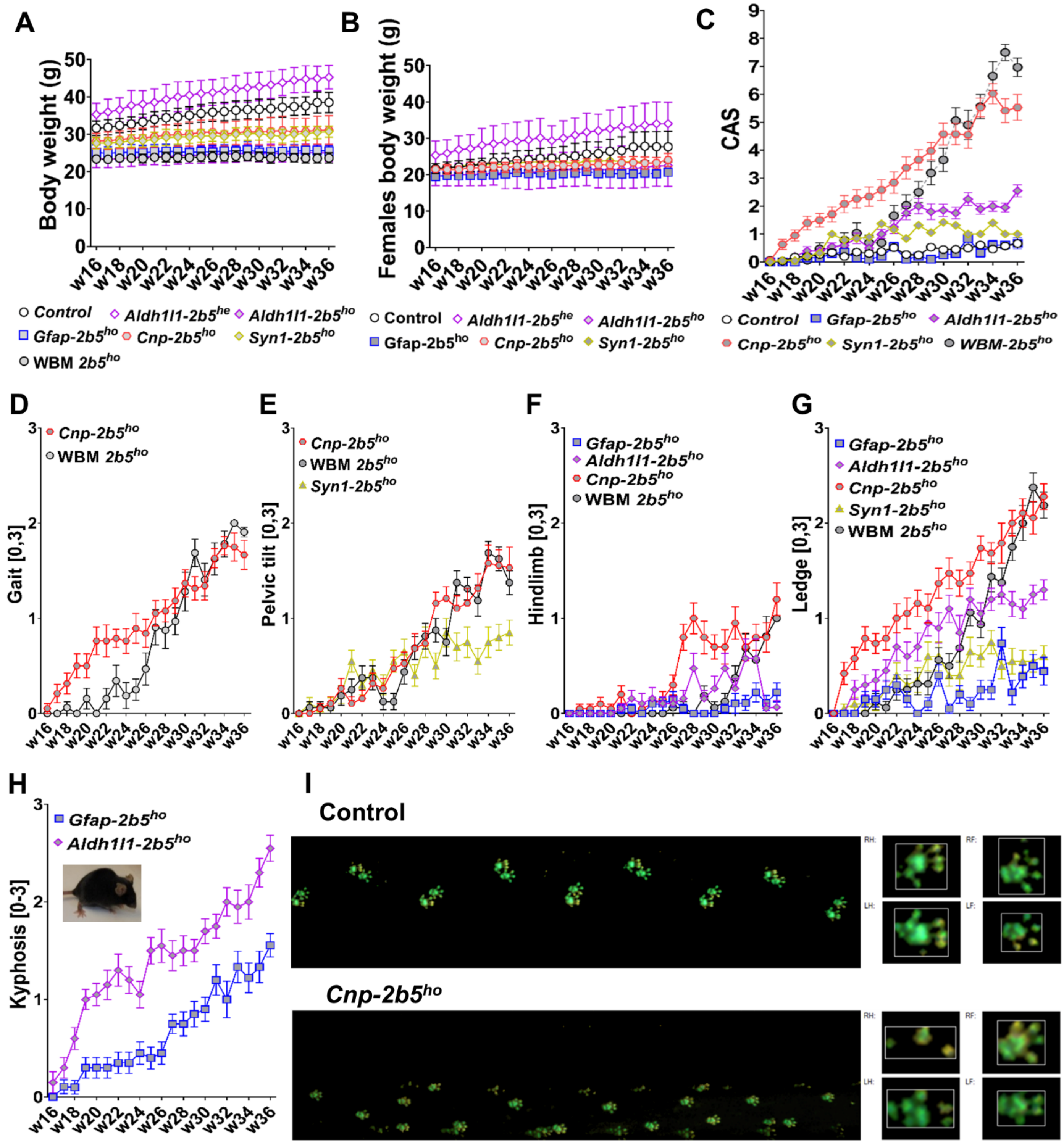

Supplementary Figure 3 Motor evaluation of the mouse models. Control group n=40, each conditional *2b5* group n=10, WBM *2b5*^{ho} n=16. **(A)** Males body weight. **(B)** Females body weight. **(C)** Composite Ataxia Score, which is a summatory of the **(D)** gait, **(E)** pelvic tilt, **(F)** hindlimb, and **(G)** ledge test. **(H)** Kyphosis was only reported in the astrocyte-specific *Eif2b5* mutant lines with and increased severity in the *Aldh1l1-2b5*^{ho} line compared to the *Gfap-2b5*^{ho} line. **(I)** Representative pawprints obtained in the CatWalk test by a control mouse (top) and a *Cnp-2b5*^{ho} mouse (bottom).

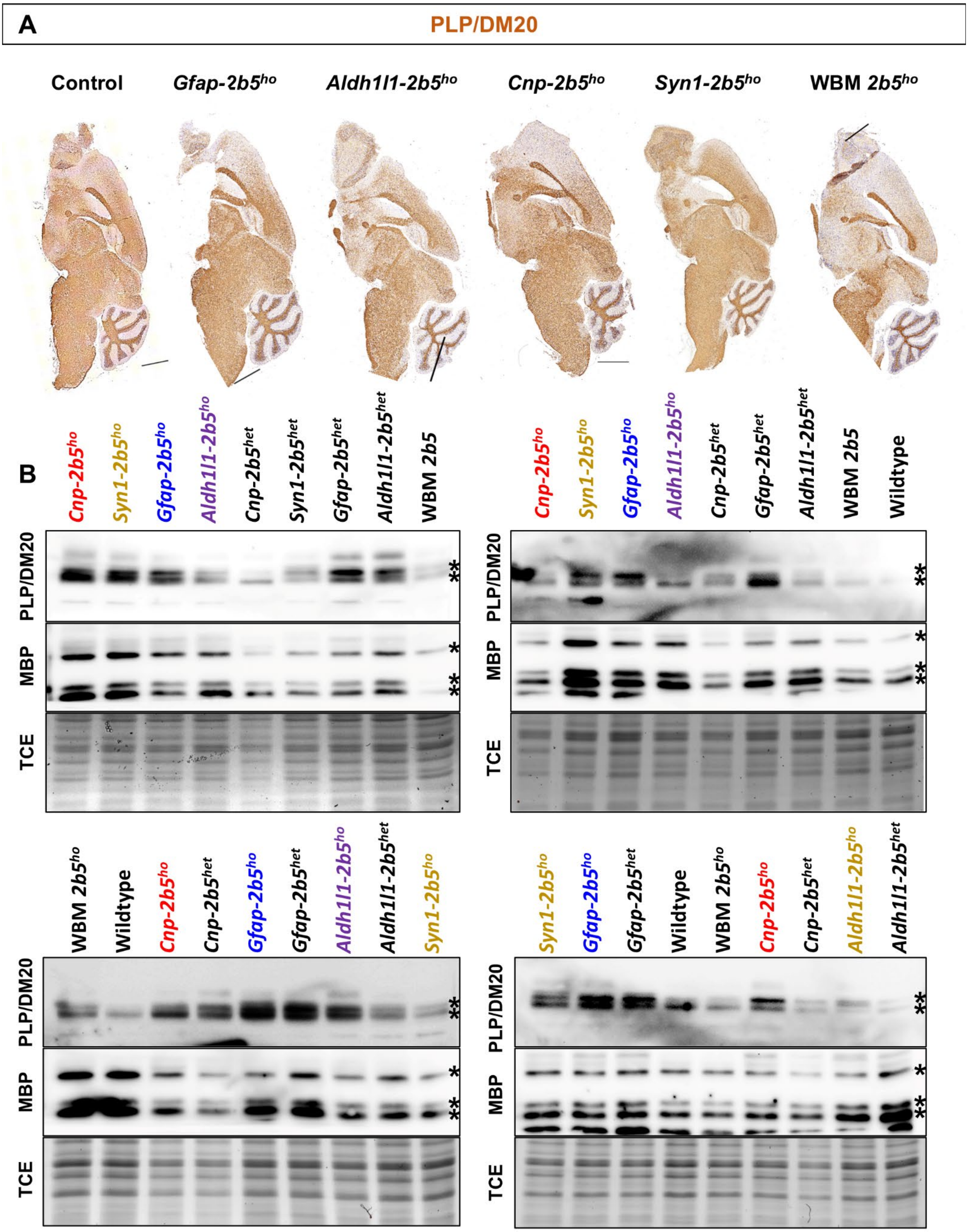

Supplementary Figure 4 Evaluation of Myelin. (A) Representative images of the PLP immunostaining in whole brain sections of conditional and WBM *2b5^ho* animals. **(B)** Western-blot images for PLP/DM20 and MBP performed in 16 control mice, 4 conditional homozygous mutant mice per line and 2 WBM *2b5^ho* mice. Asterisks represent the bands used for quantification.

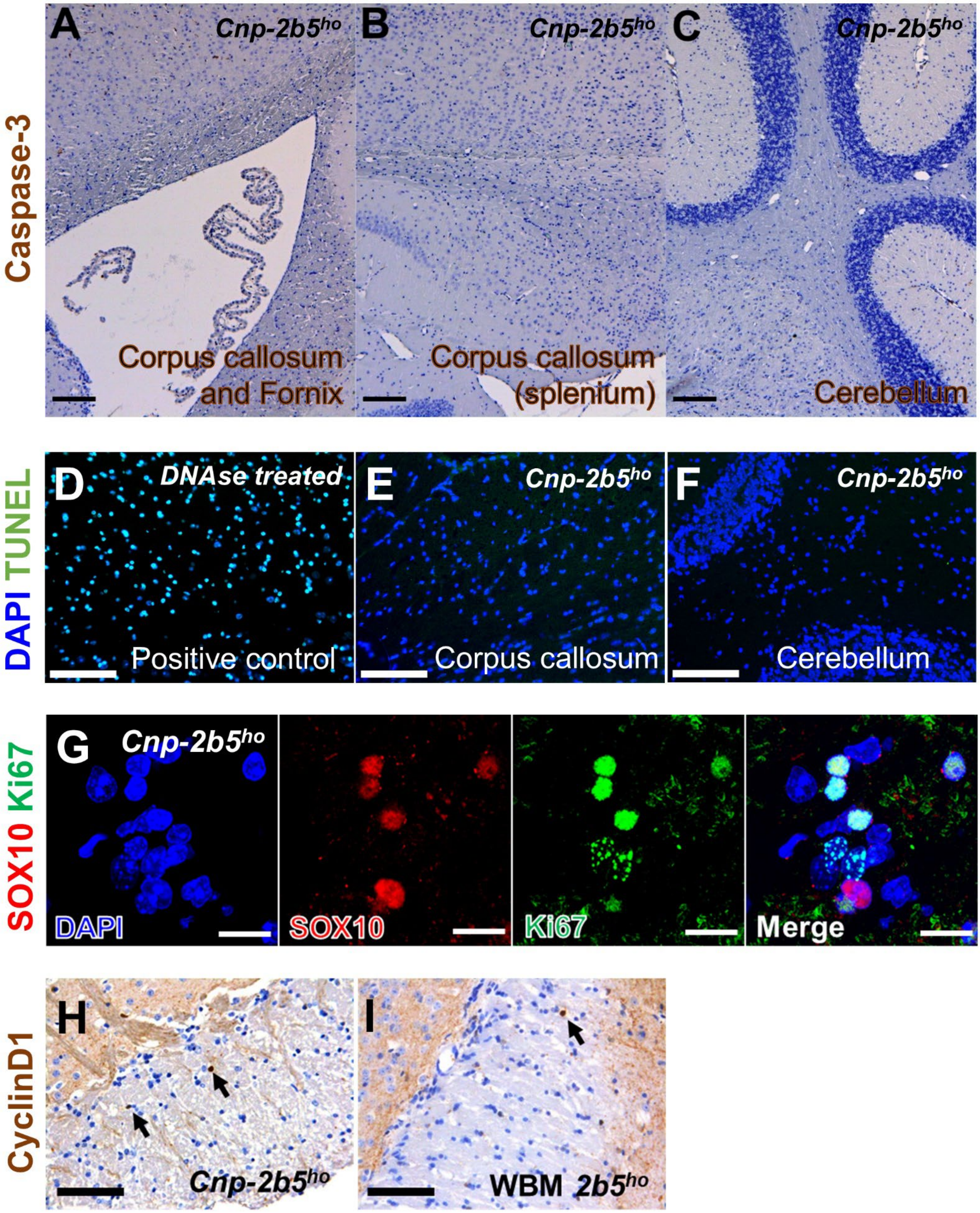

Supplementary Figure 5 Evaluation of Cell Death and Cell Cycle. **(A-C)** Representative images of the staining against Caspase-3 in one *Cnp-2b5^{ho}* mouse showing negative staining in **(A)** frontal corpus callosum and fornix, **(B)** splenium, and **(C)** cerebellar white matter. 6 controls, 6 conditional homozygous, and 6 WBM *2b5^{ho}* mice were stained. No relevant Caspase-3 positivity was detected in the white matter of any animal. **(D)** A DNAse treated sample was used as positive control for TUNEL assay (green) and counterstained with DAPI (blue). **(E,F)** All other samples showed negative TUNEL staining. **(E)** Representative image of the TUNEL assay in the corpus callosum in one *Cnp-2b5^{ho}* mouse. 6 controls, 6 conditional homozygous, and 6 WBM *2b5^{ho}* mice were stained. No TUNEL positivity was detected

in the white matter of any animal. **(F)** Representative image of the TUNEL assay in the cerebellum in one *Cnp-2b5*^{ho} mouse. 6 controls, 6 conditional homozygous, and 6 WBM *2b5*^{ho} mice were stained. No TUNEL positivity was detected in the white matter of any animal. Scale bars 100 µm. **(G)** Representative confocal images of the staining against DAPI (blue), SOX10 (red) and Ki67 (green) in the corpus callosum of *Cnp-2b5*^{ho}. SOX10^{-} cells express Ki67 in the heterochromatin (identified by the dense DAPI staining), which is indicative of cycling cells. SOX10^{+} cells show a more diffuse and homogeneous nuclear distribution of Ki67, which is characteristic of G2/prophase. Scale bar 20 µm. **(H-I)** CyclinD1 immunostaining in the **(H)** *Cnp-2b5*^{ho} and **(I)** WBM *2b5*^{ho} showed only few positive cells in the white matter of the corpus callosum. The lack of CyclinD1 staining, together with the homogeneous nuclear distribution of Ki67, may be indicative of cell cycle arrest.

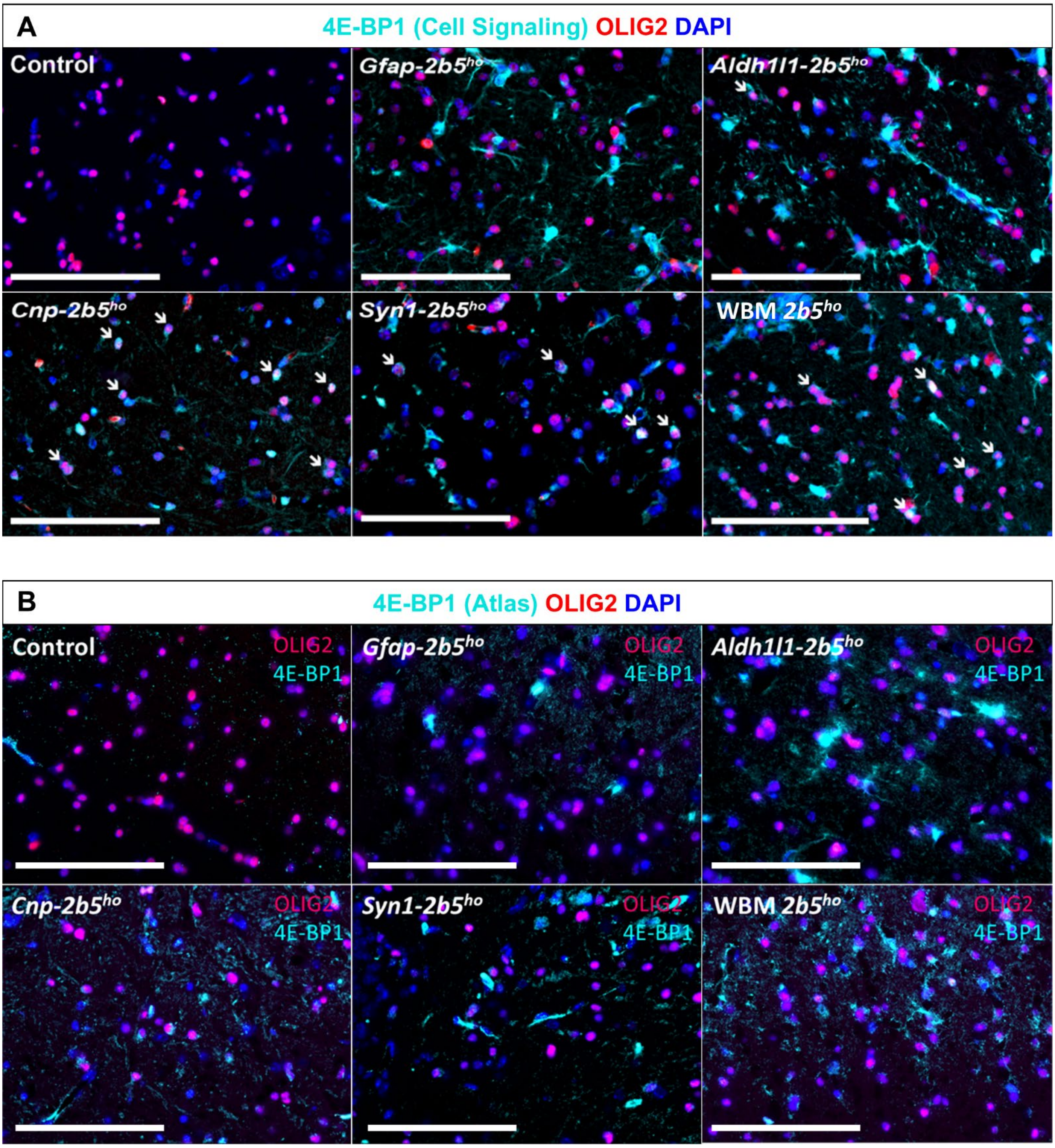

Supplementary Figure 6 4E-BP1 immunostaining in oligodendrocytes in the corpus callosum. (A) Immunostaining against 4E-BP1 (cyan) performed with the Cell Signaling antibody 9644S in combination with OLIG2 (red) and counter stained with DAPI (blue). **(B)** Immunostaining against 4E-BP1 (cyan) performed with the Atlas antibody HPA023501 in combination with OLIG2 (red) and counter stained with DAPI (blue). Both antibodies showed positivity in all homozygous mutant mice. Scale bars 100 µm.

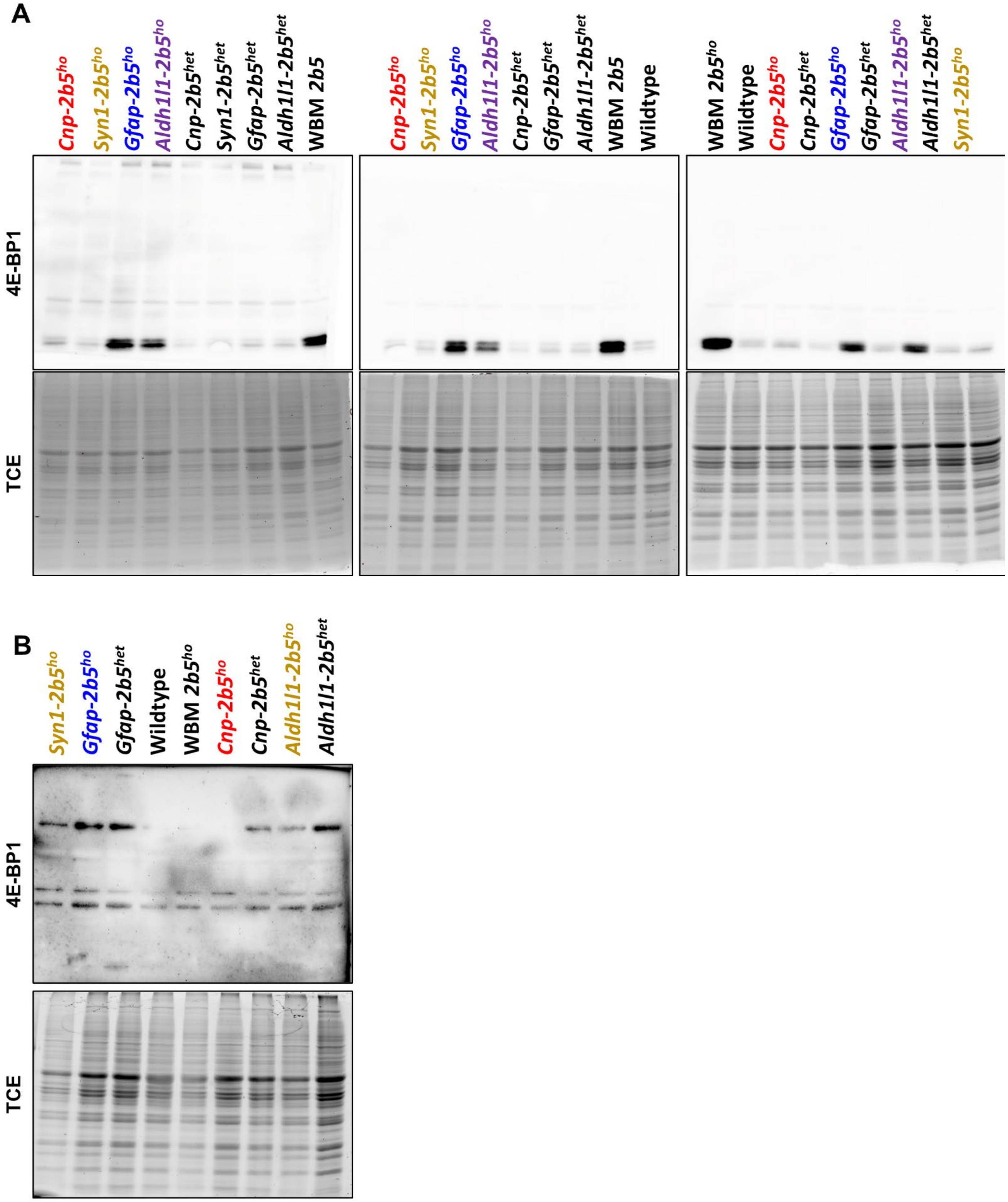

Supplementary Figure 7 4E-BP1 immunoblots. (A) Western-blot images against 4E-BP1 performed in 12 control mice, 3 conditional homozygous mutant mice per line and 2 WBM *2b5*^{ho} mice with the Cell Signaling antibody 9644S. (B) Representative image of a western blot against 4E-BP1 performed with the Atlas antibody HPA023501.
