# Supplementary material for: Cell-specific Eif2b5 mutant mice: novel insights into roles of macroglia in vanishing white matter
Source: Brain. 2025 May 6;148(11):4112–26. doi: 10.1093/brain/awaf171 (PMC12588717; doi:10.1093/brain/awaf171)

Full length gels and blots used to make the images

Figure 8B

Blot against 4E-BP1

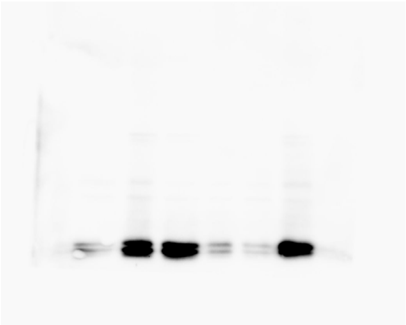

TCE gel

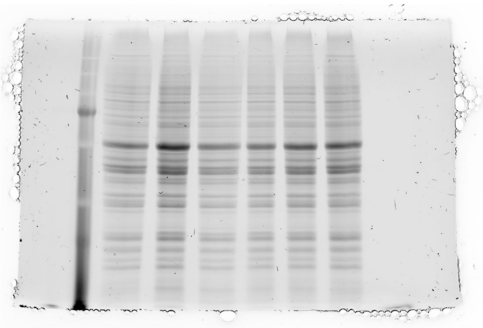

Supplementary Figure 1

Supplementary Figure 1C:

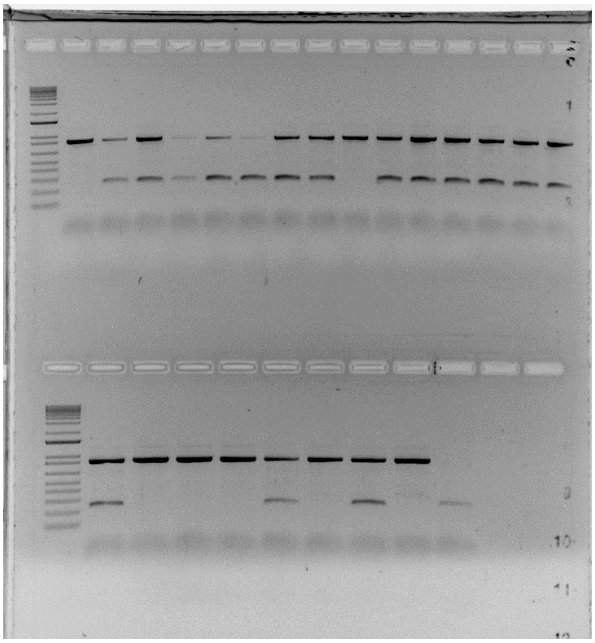

Supplementary Figure 1D:

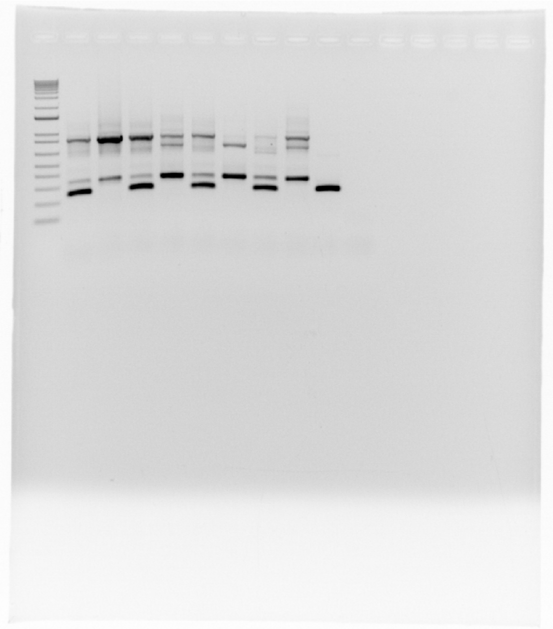

Supplementary Figure 1E:

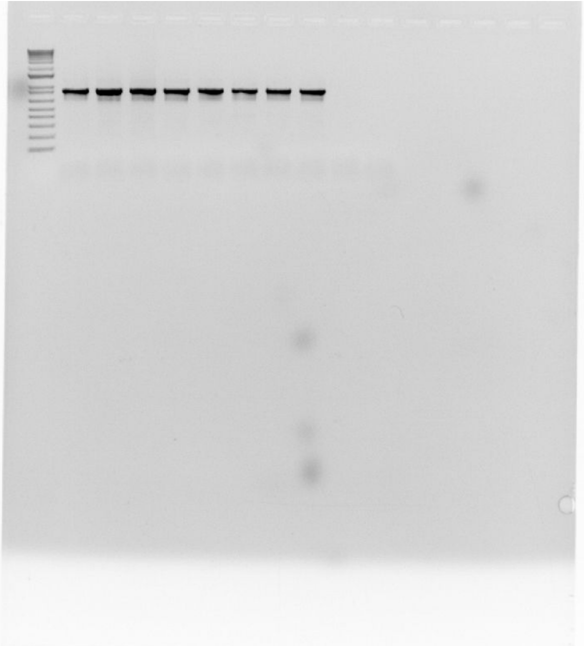

Supplementary Figure 1G:

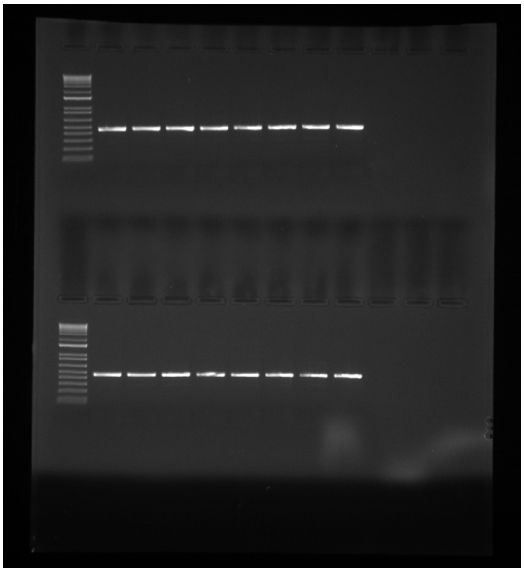

Full length images

Supplementary Figure 4B

Blot against PLP/DM20 (top left panel)

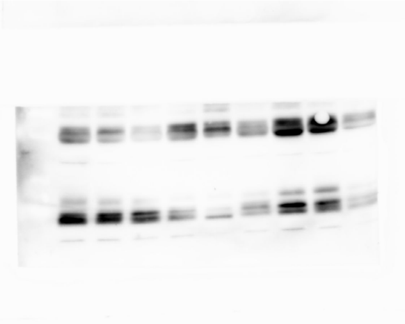

Blot against MBP (top left panel)

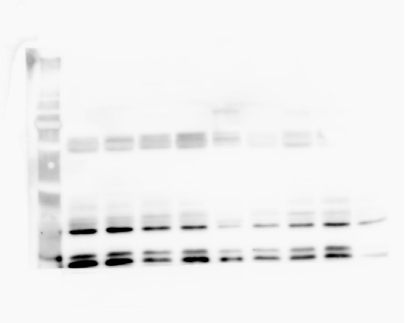

TCE gel (top left panel)

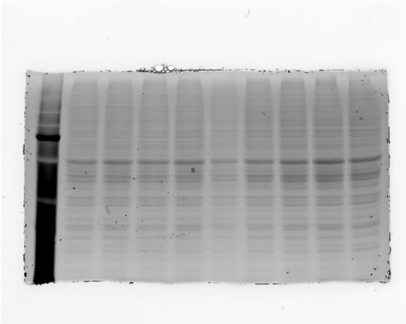

Blot against PLP/DM20 (top right panel)

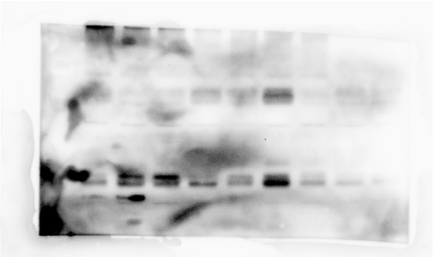

Blot against MBP (top right panel)

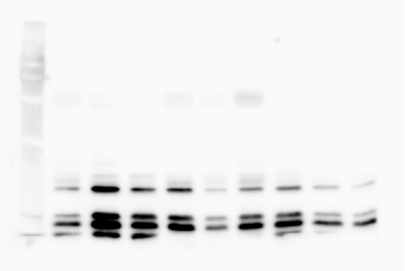

TCE gel (top right panel)

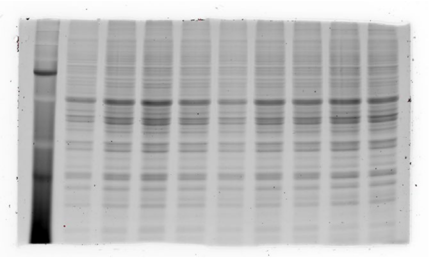

Blot against PLP/DM20 (bottom left panel)

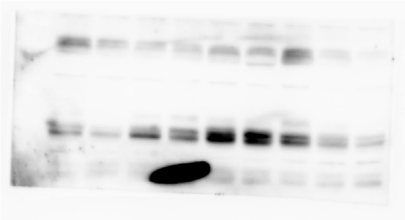

Blot against MBP (bottom left panel)

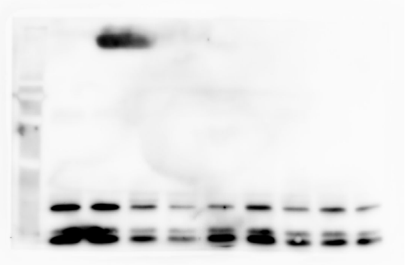

TCE gel showed in (bottom left panel)

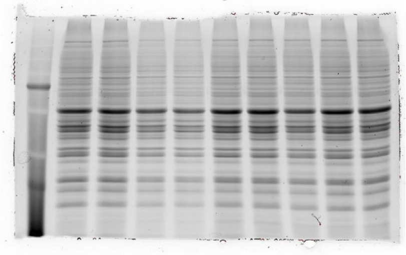

Blot against PLP/DM20 (bottom right panel)

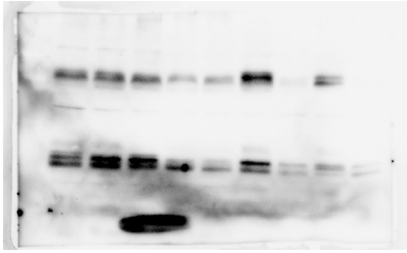

Blot against MBP (bottom right panel)

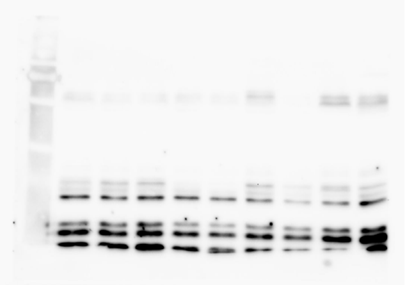

TCE gel (bottom right panel)

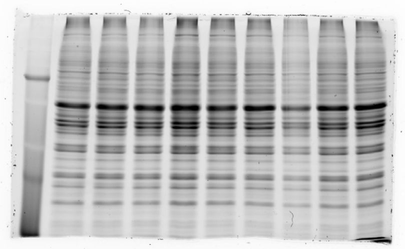

Supplementary Figure 7A

Blot against 4E-BP1(left panel)

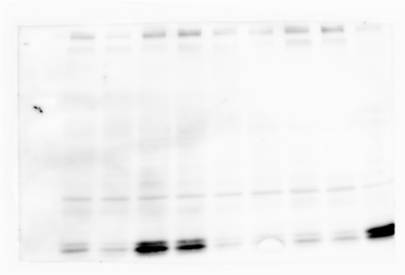

Blot against 4E-BP1(middle panel)

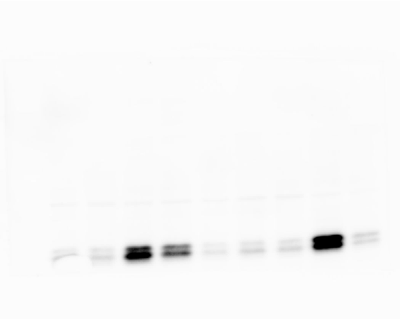

Blot against 4E-BP1(right panel)

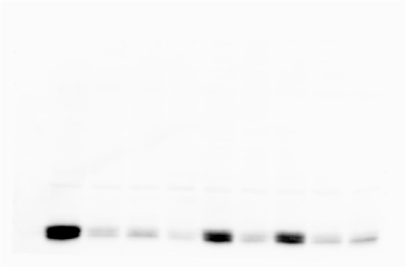

TCE gel (left panel)

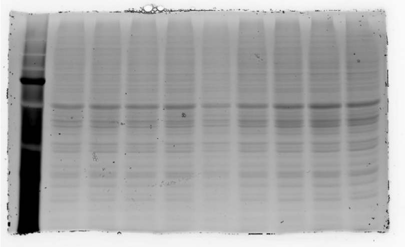

TCE gel (middle panel)

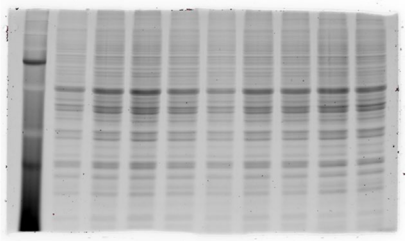

TCE gel (right panel)

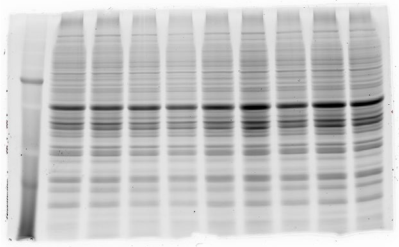

Supplementary Figure 7B

Blot against 4E-BP1

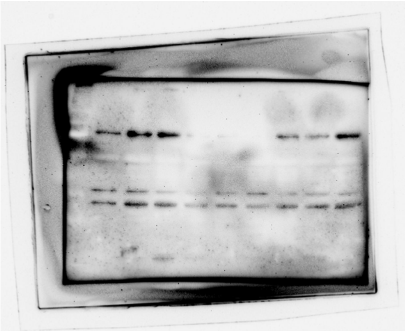

TCE gel

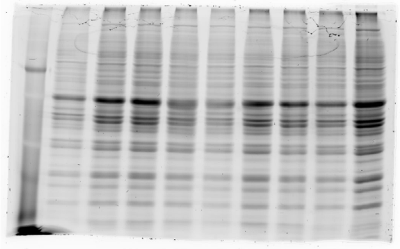

Supplement: awaf171_Supplementary_Data [file awaf171_supplementary_data.zip › brain-2024-02266-File011.pdf]
